# Folic Acid Fortification Prevents Morphological and Behavioral Consequences of X-Ray Exposure During Neurulation

**DOI:** 10.3389/fnbeh.2020.609660

**Published:** 2021-01-08

**Authors:** Kai Craenen, Mieke Verslegers, Zsuzsanna Callaerts-Vegh, Livine Craeghs, Jasmine Buset, Kristof Govaerts, Mieke Neefs, Willy Gsell, Sarah Baatout, Rudi D'Hooge, Uwe Himmelreich, Lieve Moons, Mohammed Abderrafi Benotmane

**Affiliations:** ^1^Radiobiology Unit, Interdisciplinary Biosciences, Institute for Environment, Health and Safety, Belgian Nuclear Research Centre (Studiecentrum voor Kernenergie; Centre d'étude de l'énergie nucléaire), Mol, Belgium; ^2^Laboratory of Neural Circuit Development and Regeneration, Animal Physiology and Neurobiology Section, Department of Biology, Faculty of Science, Katholieke Universiteit Leuven, Leuven, Belgium; ^3^Laboratory of Biological Psychology, Faculty of Psychology and Educational Sciences, Katholieke Universiteit Leuven, Leuven, Belgium; ^4^Molecular Small Animal Imaging Center, Biomedical MRI Unit, Department of Imaging and Pathology, Katholieke Universiteit Leuven, Leuven, Belgium

**Keywords:** radiation, radioprotectant, folic acid, birth defect, anophthalmos, agnathia, exencephaly, hyposmia

## Abstract

Previous studies suggested a causal link between pre-natal exposure to ionizing radiation and birth defects such as microphthalmos and exencephaly. In mice, these defects arise primarily after high-dose X-irradiation during early neurulation. However, the impact of sublethal (low) X-ray doses during this early developmental time window on adult behavior and morphology of central nervous system structures is not known. In addition, the efficacy of folic acid (FA) in preventing radiation-induced birth defects and persistent radiation-induced anomalies has remained unexplored. To assess the efficacy of FA in preventing radiation-induced defects, pregnant C57BL6/J mice were X-irradiated at embryonic day (E)7.5 and were fed FA-fortified food. FA partially prevented radiation-induced (1.0 Gy) anophthalmos, exencephaly and gastroschisis at E18, and reduced the number of pre-natal deaths, fetal weight loss and defects in the cervical vertebrae resulting from irradiation. Furthermore, FA food fortification counteracted radiation-induced impairments in vision and olfaction, which were evidenced after exposure to doses ≥0.1 Gy. These findings coincided with the observation of a reduction in thickness of the retinal ganglion cell and nerve fiber layer, and a decreased axial length of the eye following exposure to 0.5 Gy. Finally, MRI studies revealed a volumetric decrease of the hippocampus, striatum, thalamus, midbrain and pons following 0.5 Gy irradiation, which could be partially ameliorated after FA food fortification. Altogether, our study is the first to offer detailed insights into the long-term consequences of X-ray exposure during neurulation, and supports the use of FA as a radioprotectant and antiteratogen to counter the detrimental effects of X-ray exposure during this crucial period of gestation.

## Introduction

Exposure to ionizing radiation during embryonic development has been linked to an increased risk of birth defects. The type and severity of these defect are predominantly determined by the developmental stage during which exposure occurred (Craenen et al., 2017). Epidemiological studies on Ukrainian cohorts illustrated an increased prevalence of neural tube defects (NTDs) and eye defects (EDs) in regions severely contaminated with radioactive Cs-137 isotopes following the Chernobyl nuclear accident. Although there are no accurate dose estimates, uptake of radioactive isotopes is known to be particularly high in pregnant women living in these regions (Wertelecki et al., 2016). Initially, it was observed that the more recent Fukushima Daiichi nuclear power plant accident elicited no increase in birth defects and pre-natal mortality due to environmental radioisotope contamination (Fujimori et al., 2014), but subsequent papers debated this conclusion (Mangano and Sherman, 2015; Scherb et al., 2016). In contrast to these more recent observations, reports after the atomic bombings in Japan only mentioned an increased incidence of microcephaly and intellectual disability (Plummer, 1952; Neel and Schull, 1956). It is likely that the discrepancy in health effects of the nuclear accidents and atomic bombings stems from differences in dose, dose rate, exposure duration and radiation type. The above highlights the need to increase our knowledge about the effects of pre-natal irradiation on biological structures and functions.

Exposure to ionizing radiation during pregnancy most commonly occurs during clinical radiodiagnostic or therapeutic procedures (Mettler et al., 2009). Although medical practitioners advise against irradiation during pregnancy, it may be unavoidable in medical urgencies (Lazarus et al., 2009). In terms of radiation protection, conventional shielding methods are currently being used to partially mitigate the fetal dose (Chatterson et al., 2014; Moore et al., 2015; Owrangi et al., 2016). However, depending on the dose or the developmental stage during which exposure occurs, these conventional shielding strategies may not suffice. Animal studies have shown that the neurulation period in the early embryo is especially radiosensitive with regard to the pathogenesis of radiation-induced NTDs and ED (Russell, 1950, 1956; Di Majo et al., 1981; Heyer et al., 2000; Craenen et al., 2017, 2020b), but also in terms of cognitive disabilities and altered vision. Indeed, a decreased visual acuity in atomic-bomb survivors, irradiated in the first trimester, and born from mothers with acute radiation syndrome (≤ 2 km from hypocenter) has been reported (Burrow et al., 1964). Yet, most experimental work has focused on health risks after radiation exposure during neurogenesis, coinciding with the second trimester of human pregnancy (Plummer, 1952; Neel and Schull, 1956; Verreet et al., 2015, 2016a,b). Furthermore, there are currently no anti-teratogens or radio-protectants available to prevent (congenital) morphological and functional defects that arise from irradiation during brain development.

Folic acid (FA), a synthetic vitamin, is generally known to prevent NTDs [reviewed in Imbard et al. (2013)], in addition to other defects such as heart defects and some skeletal defects (Kappen, 2013). Besides, FA has been suggested to prevent the development of age-related neurodegenerative diseases and overall cognition (Craenen et al., 2020a). Several countries enforce staple food fortification, whereas others support FA supplementation during pregnancy (Imbard et al., 2013). Of note is that FA supplementation/fortification initiatives are currently lacking in high-risk areas, such as those severely contaminated with radioisotopes from the Chernobyl disaster. Although FA food fortification can prevent some defects such as NTDs, its efficacy depends on the causative teratogens or mutations. For example, BMS-189453 (a synthetic retinoid) causes anomalies such as NTDs and heart defects that can be prevented with FA fortification (Cipollone et al., 2009), whereas arsenate-induced NTDs do not appear to be responsive (Ferm and Hanlon, 1986). Interestingly, many of the hallmark consequences of ionizing radiation exposure, including oxidative stress, DNA damage, cell cycle arrest, cell death and epigenetic alterations, might be countered by FA (Heyer et al., 2000; Martin et al., 2014; Reisz et al., 2014).

This study is the first to offer an in-depth analysis of the morphological and behavioral consequences of irradiation during neurulation in mice. To this end, we used a multidisciplinary approach, including an extensive behavioral test battery and imaging techniques such as spectral domain optical coherence tomography (SD-OCT) and magnetic resonance imaging (MRI). In addition, we assessed the efficacy of FA food fortification in preventing fetal malformations as well as adult functional and morphological defects resulting from X-ray exposure.

## Materials and Methods

### Animals and FA Fortification

All animal experiments were conducted in line with the relevant guidelines and were approved by the Institutional Ethical Committees of SCK-CEN/VITO (ref. 02–012) and the Animal Welfare Committee of the KU Leuven University, and are in strict accordance with the European Communities Council Directive of 22 September 2010 (2010/63/EU). C57BL6/J mice (Janvier, Bio Services, The Netherlands) were housed in individually ventilated cages, under standard laboratory conditions (12-h light/dark cycle) and fed *ad libitum*. One week before coupling, animals designated for the macroscopic fetal study were placed on a control Teklad (Carfil Quality, Oud-Turnhout, Belgium) diet (3.5 mg/kg FA), a FA fortified diet (8 mg/kg FA) or an extra-FA fortified diet (12 mg/kg FA). The FA concentrations within the final customized food products were investigated in compliance with ISO 17025. We selected the dose of 8 mg/kg because it was observed that this is an effective concentration to achieve antiteratogenic effects in mice (Gray and Ross, 2009; Harris, 2009). A dose of 12 mg/kg was included based on the assumption that some teratogens require higher doses of FA (Gray and Ross, 2009; Harris, 2009).

Animals designated for the behavioral tests and MRI were limited to the control diet or the 8 mg/kg FA diet, and were kept on their respective diets until they were euthanized. Timed couplings were performed during a 2-h period at the start of the light phase (7:30 a.m.−9:30 a.m.) to attain synchronous timing of embryonic development. The day of coupling was identified as E0. At E7.5, animals were placed in a Plexiglas holder and transported to the irradiation installation. Mice intended for the macroscopic study were either sham-irradiated or irradiated with 1.0 Gy of X-rays. Animals used for behavioral testing and MRI were sham-irradiated or received a sub-lethal dose of 0.1 or 0.5 Gy of X-rays at E7.5. Irradiation was performed using an X-strahl 320 kV (0.14 Gy/min, inherent filtration: 0.21 mmAl, additional filtration: 3.8 mm Al + 1.4 mm Cu + DAP, tube voltage: 250 kV, tube current: 12 mA,) in accordance to ISO 4037. The number of animals used for the macroscopic, skeletal, behavioral and MRI experiments is depicted in Table 1, unless otherwise specified.

**Table 1:** Sample sizes.

	**Control diet**								**High FA diet (8 mg/kg)**								**High FA diet (12 mg/kg)**	
	**0.0 Gy**		**0.1 Gy**		**0.5 Gy**		**1.0 Gy**		**0.0 Gy**		**0.1 Gy**		**0.5 Gy**		**1.0 Gy**		**1.0 Gy**	
	***n***	***N***	***n***	***N***	***n***	***N***	***n***	***N***	***n***	***N***	***n***	***N***	***n***	***N***	***n***	***N***	***n***	***N***
Macroscopic	126	15	n.a.		n.a.		116	18	n.a.		n.a.		n.a.		114	19	107	16
Skeletal	9	3	n.a.		n.a.		9	3	n.a.		n.a.		n.a.		9	3	9	3
Behavior/OCT	10	4	13	6	12	6	n.a.		12	4	11	6	12	6	n.a.		n.a.	
MRI	9	4	12	6	5	3	n.a.		5	2	7	5	7	4	n.a.		n.a.	

*N, number of litters; n, number of fetuses*.

### Macroscopic Scoring and Skeletal Stainings

The dissections, macroscopic scorings and alcian blue/alizarin red skeletal stainings were performed at E18 as previously described (Craenen et al., 2017). For the skeletal analyses, E18 fetuses were randomly selected from the macroscopic study. The axial skeleton was analyzed, with a focus on the vertebrae and the ribs. A subdivision was made between atlas, cervical, thoracic, lumbar, sacral and caudal vertebrae, whilst also differentiating between true, false and floating ribs and sternum.

### Behavioral Tests

Starting at week (W)5 and ending at W14, behavioral tests were performed on male mice in the order described below (Table 2). All experiments were performed under blinded conditions. To assess visual acuity, optokinetic tracking was performed. We included cage activity to assess global activity, during both light and dark-phase, and assessed explorative and social behavior with the open field and social exploration tests. The elevated plus maze (EPM) was included to ascertain anxiety, whereas the accelerating rotarod was used to identify issues in motility. Next, to explore olfactory performance we used the odor habituation/dis-habituation assay. Finally, two tests for memory were included: the Morris water maze (MWM) and passive avoidance, to test spatial and fear-related memory, respectively.

**Table 2:** Overview of test order and age at time of testing.

**Protocol**	**Age range (weeks)**
Optokinetic tracking response	W5–W7
Optical coherence tomography	W5–W7
Cage activity	W7–W9
Open field	W7–W9
Social exploration	W8–W10
Elevated plus maze	W8–W10
Accelerating rotarod	W8–W10
Odor habituation/dis-habituation	W9–W11
MRI	W9–W11
Morris Water Maze	W10–W13
Passive avoidance	W12–W14

#### Optokinetic Tracking Response

Using a virtual-reality chamber (OptoMotry, Cerebral Mechanics, Medicine Hat, AB, Canada), the optokinetic tracking response was assessed (De Groef et al., 2016; Van Hove et al., 2016). The animal was placed on the center of an elevated platform within the optokinetic installation, where a vertical sine wave pattern was displayed on the monitors. Using a real-time camera system, visual acuity was scored manually using a staircase procedure, composed of random spatial frequencies (100% contrast, 12° per second speed).

#### Cage Activity

The impact of ionizing radiation exposure on ambulatory behavior was investigated over a 23 h time-period, starting at 4 p.m. until 3:30 p.m. the next day (Verreet et al., 2016a). During this period, animals were individually housed in transparent cages (20 × 26 cm) with minimal bedding, chow and water and placed in a laboratory-built activity logger with three infrared beams. Beam breaks were recorded over 30 min time bins.

#### Open Field and Social Exploration

To assess exploration and social interaction, a transparent Plexiglas arena (50 × 50 cm) was used (Stroobants et al., 2008; Bollen et al., 2015; Callaerts-Vegh et al., 2015). The arena was homogenously illuminated and equipped with an Any-maze (Dublin, Ireland) tracking system. For the open field test, animals were placed in the empty arena for 1 min of acclimatization, immediately followed by a 10 min test phase with active tracking. The social exploration experiment was identical to the open field test, except that in the center of the arena a small cage with two same-sex strange mice was placed.

#### Elevated Plus Maze

In order to investigate anxiety, animals were subjected to EPM testing as was previously described (Verreet et al., 2015). The cross-shaped EPM consisted of two perpendicular open and closed arms (21 × 5 cm). Five infrared detectors were installed on the EPM: 2 at the exits out of the closed arms and two at the entrances to the open arms (entries/exits) and one along the length of the open arms (time spent on the open section). The animal was placed in a closed arm and after 1 min of adaptation, beam breaks were recorded for 10 min.

#### Accelerating Rotarod

General motor function and balance following *in utero* X-ray exposure during neurulation were assessed using an accelerating rotarod (Ugo Basile, Italy), as was described previously (Verreet et al., 2015). Initially, the animals underwent two adaptation trials (2 min each), each at a constant speed of 4 rpm. In turn, the mouse was subjected to four subsequent test trials, where during each 5 min trial the rotation speed gradually increased from 4 to 40 rpm. Latency was recorded when the mouse lost its footing and fell off the rotating beam.

#### Odor Habituation and Dis-Habituation

To assess the interaction of mice with olfactory cues, i.e., habituation and dis-habituation, the animals were subjected to an odor discrimination test as was described previously (Yang and Crawley, 2009; Yang et al., 2012; Arbuckle et al., 2015). Animals were individually placed in a fresh cage with a small amount of bedding, followed by 30 min of acclimatization with a dry cotton swab fixed to the cover grid (tip ~5 cm from bottom). Next, the animals were exposed to a sequence of 15 subsequent odor exposures (2 min each): Three trials with water, three trials with grape (non-social odor 1 = NS1), three trials with banana (NS2), three trials with social odor one (S1) and three trials with social odor two (S2). For the preparation of the NS odor tests, respectively 1:100 diluted grape extract (SAFC, W26820-8-K methyl anthranilate ≥98%) and 1:100 diluted banana extract (Acros Organics, AC269481000 n-Butyl propionate >99%) on cotton tips was used. For the S odors, cotton tips were dipped in water and moved in a cross-pattern through the bedding of soiled cages of same-sex mice. During each trial, sniffing-time was recorded manually whenever the subject's nose was within a 2 cm radius of the cotton swab. The inter-session interval never exceeded 2 min.

#### Morris Water Maze

In order to assess whether FA and sub-lethal pre-natal doses of X-rays during neurulation affected adult spatial learning, MWM was performed. Animals were tested in a circular pool (diameter 150 cm, height 30 cm), filled with opacified non-toxic water as previously described (Latif-Hernandez et al., 2016; Verreet et al., 2016a). For the acquisition trials, a see-through acrylic platform was consistently placed in the same quadrant, 1 cm below the water surface. The pool was located in the center of a homogeneously-lit room, with invariable visual cues. Acquisition training was performed over a period of 5 days, followed by a 2-day resting period, followed again by 5 days of training. During each training day, every mouse was subjected to four trials. The trial interval was approximately 15-min and the quadrant-starting positions varied in a semi-random order for every trial. If the animal was unable to find the platform within 120 s, it was placed on the platform for 10 s and subsequently removed from the basin. On day 5 of the acquisition trials and 2 days after the last acquisition trial, probe trials were performed. During these probe trials, the platform was removed from the basin and mice were subjected to a single probe trial of 100 s, where the starting position was opposite to the target quadrant. Using an automated video capture and tracking system (EthoVision, Noldus, The Netherlands), various parameters such as trajectory and swim speed were recorded. We observed floating behavior (swim velocity <5 cm/s, more than 30 s per swim) in all groups, except the control diet + 0.0 Gy group. However, for the path length analysis to determine if the animals covered the same track during learning, we included all animals due to the low animal numbers per group. Non-responders (floating >35% of test time) were excluded for the reference memory test. As such, a reduced number of animals was included for the reference memory test, as compared to Table 1. More specifically, for this analysis in particular we included under control diet condition 29 animals (9 for 0.0 Gy, 11 for 0.1 Gy and 9 for 0.5 Gy), and 27 under high FA condition (10 for 0.0 Gy, 9 for 0.1 Gy and 8 for 0.5 Gy).

#### Passive Avoidance

We investigated fear-aggravated learning and memory using a passive avoidance set-up (Lo et al., 2013). Animals were placed in a brightly lit compartment and the door leading into a dark adjacent chamber was opened after 5 s. Latency to enter the dark chamber was timed starting immediately after opening of the dark chamber and stopped when the animal had all four paws on the electric grid in the dark room. Next, the door separating the two compartments was closed and a shock (0.3 mA, 2 s) was administered. The next day, the procedure was repeated, albeit without the administration of an electric shock.

### *In vivo* Imaging

#### Optical Coherence Tomography

To assess retinal development and thickness, SD-OCT was used as was previously discussed (Van Hove et al., 2016). The animal was anesthetized by intraperitoneal (ip) injection of 75 mg/kg body weight ketamine (Anesketin, Eurovet, Bladel, The Netherlands) and 1 mg/kg medetomidine (Domitor, Pfizer, NY, USA). Shortly before imaging, pupils were dilated using topical 0.5% tropicamide (0.5% Tropicol, Thea Pharma, Wetteren, Belgium). Next, SD-OCT was performed using an Envisu R2210 (Bioptigen, Morrisville, NC, USA) via 100 serial B-scan lines with each line consisting of 1,000 A-scans, in a 1.4 × 1.4 mm field. Afterwards, ip injection of atipamezol (1 mg/kg, Antisedan, Pfizer) was applied to reverse the anesthesia. Thickness of the retina was investigated using InVivoVue Diver software (Bioptigen).

#### Magnetic Resonance Imaging

For MRI we used female mice, which originated from the same litters as the behavioral test mice. When the female mice were on average W10, *in vivo* MR imaging of the brain was performed using a 7 T Bruker Biospec 70/30 MRI scanner (30 cm horizontal bore with actively shielded gradients (200 mT/m), Bruker Biospin, Ettlingen, Germany). All data were acquired using a quadrature volume coil (72 mm internal diameter, transmit, actively decoupled) in combination with a dedicated mouse brain surface receive coil (Bruker Biospin). To obtain high resolution 3D images of the entire mouse brain, image acquisition and animal anesthesia was performed similar to previously described experiments (Verreet et al., 2016a). In brief, after the acquisition of localizer scans morphological 3D MR imaging was performed using a rapid acquisition relaxation enhancement (RARE) T2-weighted sequence with a RARE factor of 16 and a repetition time and echo time of 1,000 ms and 67 ms, respectively. The field of view was 24 × 15 × 8.3 mm with a matrix of 256 × 160 × 88, resulting in an isotropic resolution of 94 μm. The total acquisition time was 16 min. The methodology of image post-processing and the labeled template was based on previously published work (Verreet et al., 2016a). Briefly, we first corrected for image intensity inhomogeneity using the N4 bias field correction algorithm (Tustison et al., 2010) using an in-house developed MeVislab pipeline (MeVis Medical Solutions, Germany). Images were affinely registered to the template used in Verreet et al. (2015, 2016a) to obtain brain masks for each animal, which were isotropically dilated by 2 voxels. These brain masks were applied to the raw data and the bias field correction was repeated. Finally, images were non-rigidly registered to the template using the Fast Free-Form Deformation algorithm implemented in Niftyreg (Modat et al., 2010). Template labels were propagated to the individual study images using the transformations obtained from this step, and quantified using an in-house developed Python script (Python 2.7, Python Software Foundation).

### Statistics

Statistical analyses were performed with GraphPad Prism 7.02 (GraphPad Software, San Diego, CA, USA). To analyze the data on the macroscopic and skeletal defects, the Kruskal-Wallis methodology was used, in combination with Dunn's *post-hoc* testing. Data on pre-natal viability were assessed using two-way ANOVA and Dunnet testing for multiple comparisons. For most behavioral tests, MRI and SD-OCT, we used two-way ANOVA (with pairing where required) in combination with Dunnet (inter-dose comparisons) and (Holm-)Sidak (inter-diet comparisons) *post-hoc* tests. To assess dishabituation, paired *t*-testing was done, whilst two-way ANOVA + Sidak was utilized to investigate habituation. To perform inter-dose and inter-diet comparisons, one-way ANOVA + Dunnet was used in conjunction with the first trial of the different odors. For all statistical tests, a *p*-value of 0.05 was considered statistically significant. All values are represented as mean ± SEM.

## Results

### FA Reduces the Prevalence of Radiation-Induced Anophthalmos, Exencephaly and Agnathia

**Figure 1:**
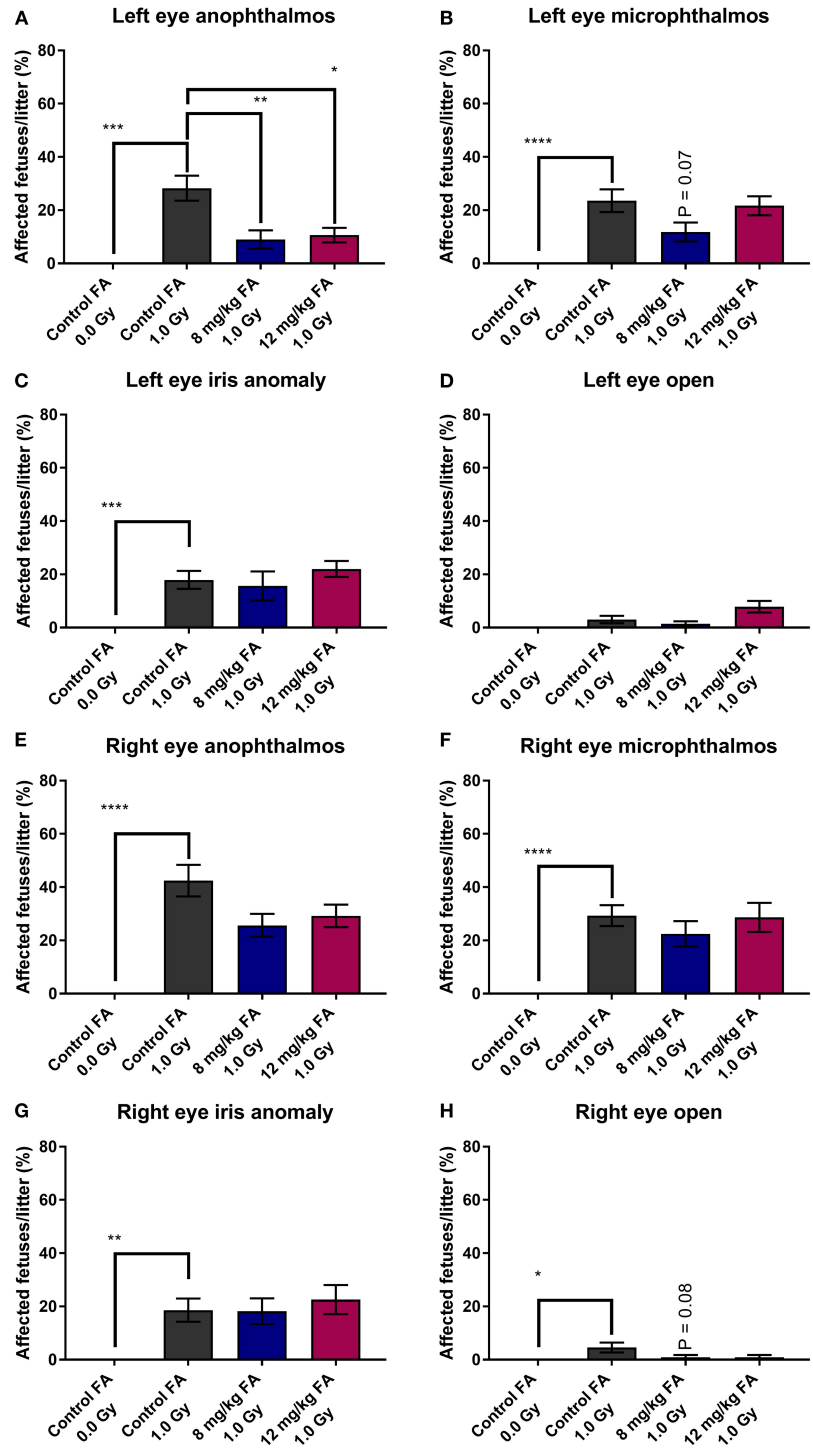
Prevalence of various eye defects observed at E18, following 1.0 Gy X-ray exposure at E7.5, and prevention with FA fortification. Radiation significantly increased the risk for left eye anophthalmos; a defect that could in turn be prevented by both FA fortified diets (8 mg/kg and 12 mg/kg FA) **(A)**. Although both left eye microphthalmos **(B)** and iris anomaly **(C)** were induced by X-irradiation, no rescue effect of FA was observed on these phenotypes. The left eye open phenotype was not increased in prevalence following irradiation **(D)**. Although defects of the right eye, including anophthalmos **(E)**, microphthalmos **(F)**, iris anomaly **(G)** and open eye **(H)** were more prevalent following irradiation, we observed no significant prevention of these defects by FA. Data are represented as mean ± SEM, ^*^p ≤ 0.05, ^**^p ≤ 0.01, ^***^p ≤ 0.001, ^****^p ≤ 0.0001.

First, we examined the prevalence of radiation-induced EDs and the prevention thereof with FA fortification. The prevalence of left-eye anophthalmos (Figure 1A), microphthalmos (Figure 1B) and iris anomaly (Figure 1C) was significantly increased following X-irradiation (respectively, 28.26 ± 4.72, 23.56 ± 4.28, and 17.92 ± 3.39 %). In contrast, X-irradiation did not affect the prevalence of the left eye open phenotype (3.03 ± 1.40%) (Figure 1D). Similar observations were made for the right eye (respectively 42.41 ± 5.93, 29.32 ± 3.93, and 18.59 ± 4.35%) (Figures 1E–H). Here, the right eye also showed an increase of the open phenotype (4.55 ± 1.89%). Of interest, we revealed a partial prevention of radiation-induced left-eye anophthalmos with both the 8 mg/kg FA (9.02 ± 3.40%) and 12 mg/kg FA (10.62 ± 2.74%) diets (Figure 1A). No such rescue was observed for the right eye (Figure 1E).

**Figure 2:**
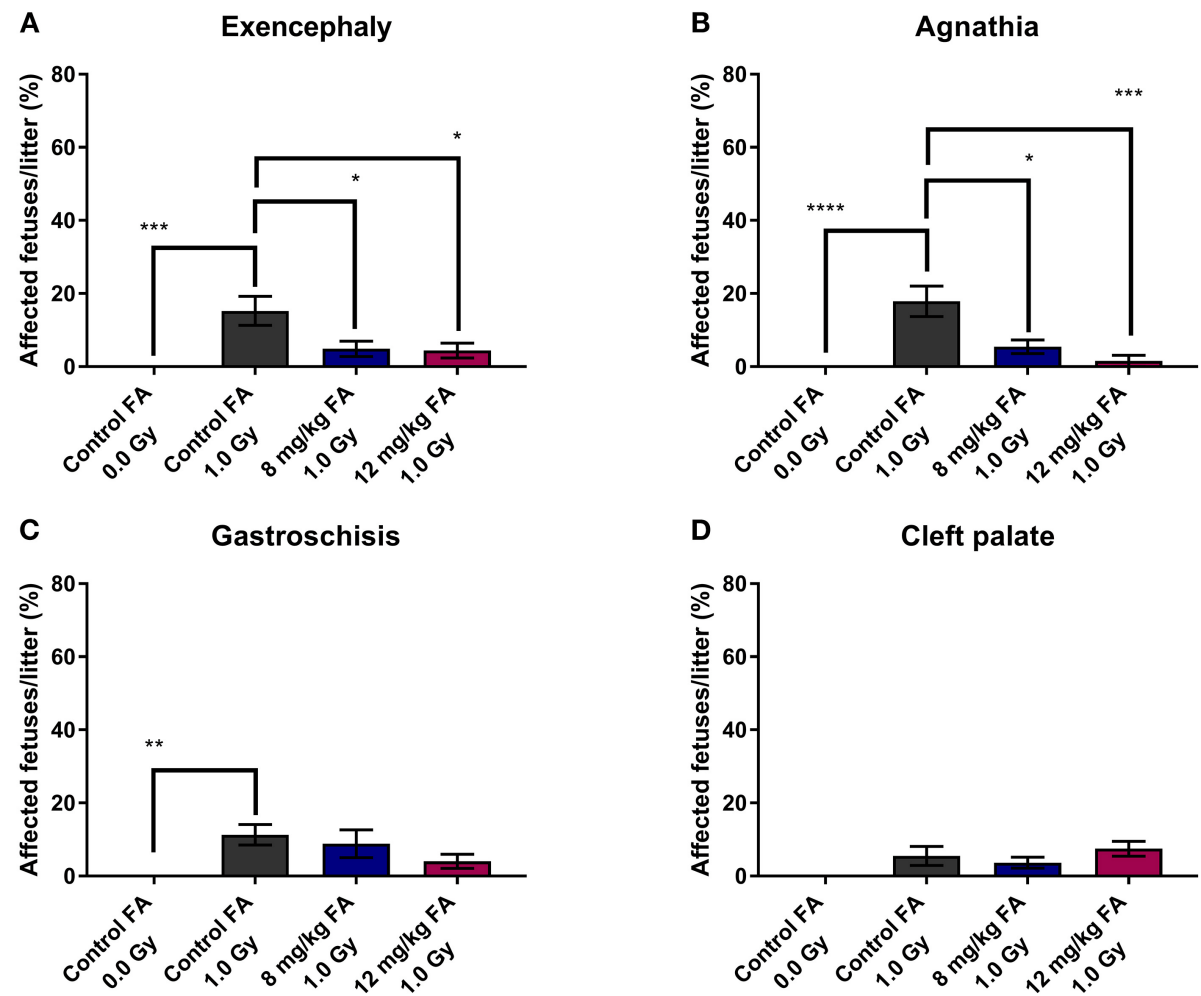
Prevalence of radiation-induced birth defects at E18, affecting the head and abdomen, and the prevention with FA. Irradiation at E7.5 significantly increased the risk for exencephaly **(A)**, agnathia **(B)** and gastroschisis **(C)**. The first two of these radiation-induced defects could be partially prevented by FA food fortification (both 8 and 12 mg/kg) **(A,B)**. Radiation had only minimal impact on the prevalence of cleft palate **(D)**. Data are represented as mean ± SEM, ^*^p ≤ 0.05, ^**^p ≤ 0.01, ^***^p ≤ 0.001, ^****^p ≤ 0.0001.

In addition, we determined the number of fetuses with exencephaly, agnathia, gastroschisis and cleft palate. X-irradiation increased the prevalence of exencephaly (15.26 ± 3.95%) and agnathia (17.88 ± 4.17%) when the animals were fed the control diet, whilst 8 and 12 mg/kg FA provided significant prevention of both exencephaly (respectively, 4.89 ± 2.10 and 4.43 ± 2.03%) (Figure 2A) and agnathia (respectively, 5.41 ± 1.86 and 1.56 ± 1.56%) (Figure 2B). Furthermore, irradiation also increased the number of fetuses affected by gastroschisis in mothers on the control diet (11.3 ± 2.83%), but here no rescue was observed with the FA fortified diets (Figure 2C). Finally, X-ray exposure at E7.5 did not affect the occurrence of cleft palate in the fetuses, regardless of the diet (Figure 2D).

### FA Counteracts the Effects of X-Ray Exposure on Pre-natal Survival

**Figure 3:**
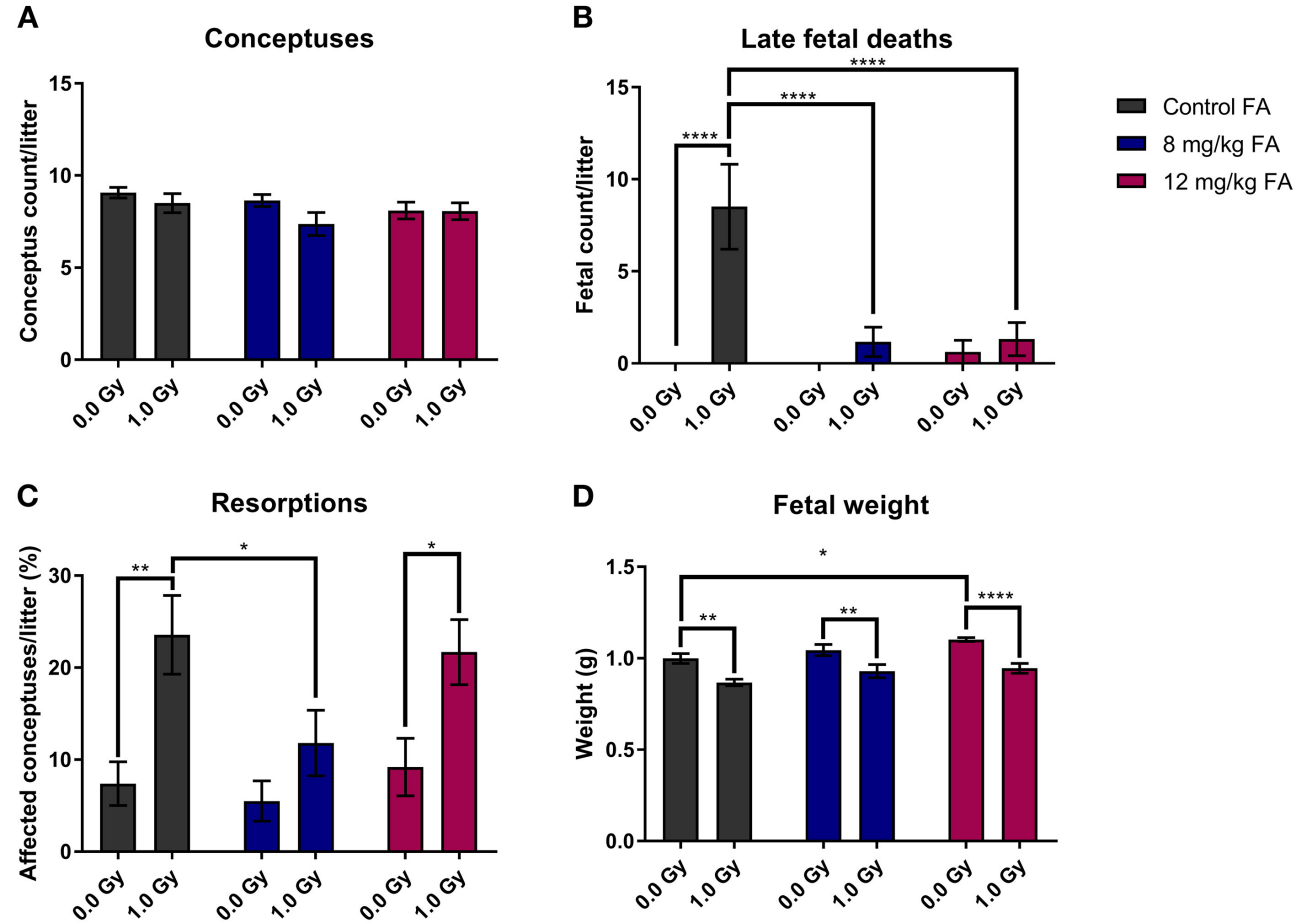
Impact of 1.0 Gy X-ray exposure at E7.5 and FA on the number of conceptuses **(A)**, late fetal deaths **(B)**, resorptions **(C)** and fetal weight **(D)**. Neither radiation nor FA diet (8 and 12 mg/kg FA) had an impact on the number of conceptuses at E18 per litter **(A)**. Irradiation strongly increased the rate of late fetal deaths when mothers were fed the control diet, whilst FA fortification prevented this **(B)**. A significant increase in resorptions was observed for irradiated mice on the control diet, which was in turn prevented only by the 12 mg/kg FA diet **(C)**. FA fortification significantly increased fetal weight, whilst irradiation decreased fetal weight **(D)**. Data are represented as mean ± SEM, ^*^p ≤ 0.05, ^**^p ≤ 0.01, ^****^p ≤ 0.0001.

In the next part of our study, we investigated the impact of X-irradiation during neurulation on the number of implants, pre-natal deaths and fetal weight. Neither X-irradiation nor FA fortification affected the total number of conceptuses per pregnant female (Figure 3A). In terms of late fetal deaths (E18 fetuses with no signs of life), an increase was observed after irradiation in mothers on the control diet, while this increase was prevented with 8 and 12 mg/kg FA diets (Figure 3B). Furthermore, we found an increase in resorptions (implantation site at E18, which holds no developed fetus, and shows evident embryonic-stage death) after 1.0 Gy irradiation, with a notable rescue after 8 mg/kg, but not after 12 mg/kg FA fortification (Figure 3C). Finally, irradiation resulted in a marked fetal weight loss at E18 (Figure 3D), which was not rescued following FA fortification. Of note, sham-irradiated fetuses gained weight when placed on the 12 mg/kg FA diet, as compared to sham-irradiated animals on the control diet (Figure 3D). Altogether, we were able to demonstrate a preventive role of FA for radiation-induced late fetal deaths and resorptions.

### Axial Skeletal Defects and Prevention With FA

**Figure 4:**
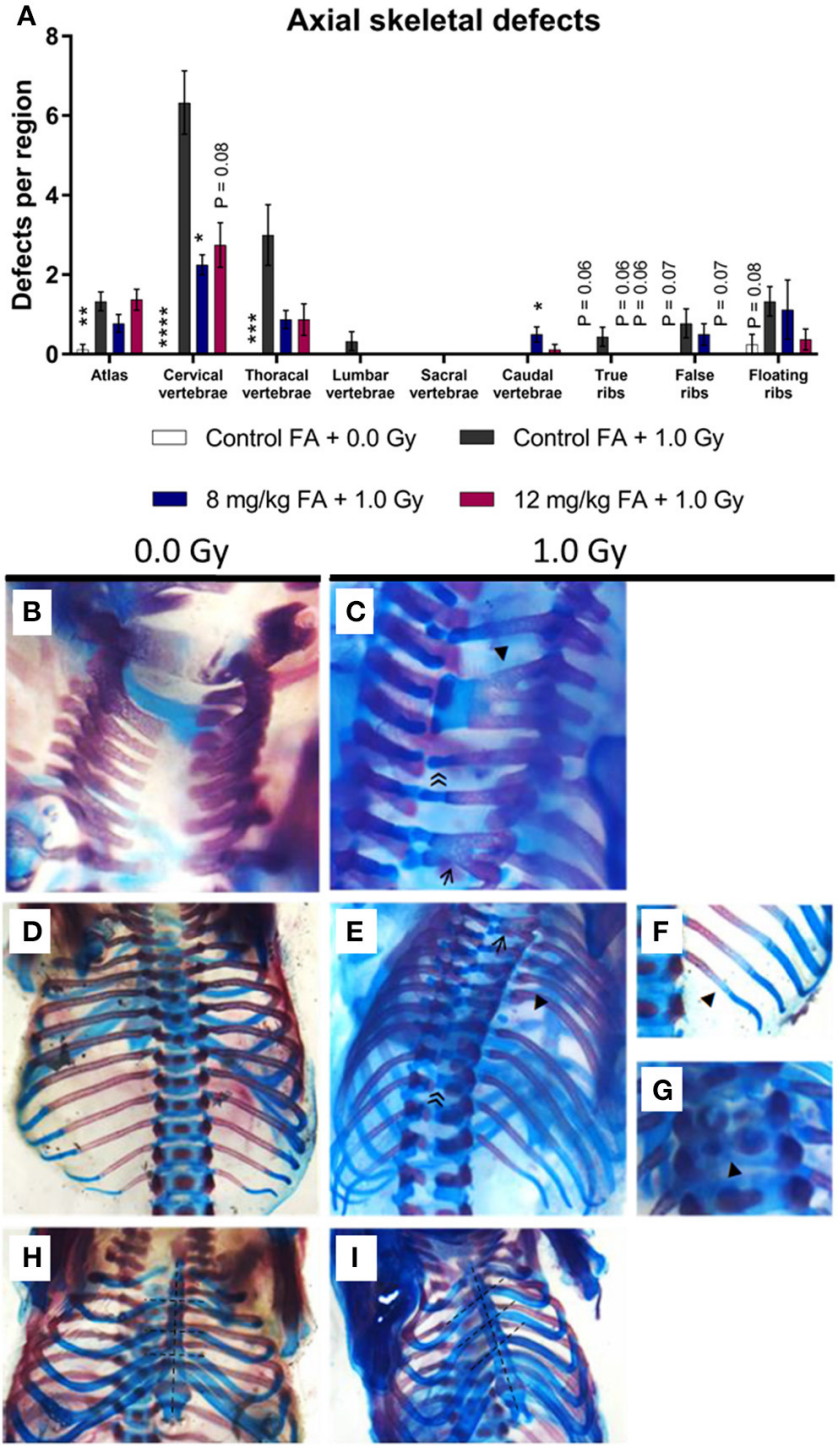
Prevalence and categories of axial skeletal defects at E18, following 1.0 Gy at E7.5 and prevention by FA. **(A)** Radiation significantly increased the number of defects in the atlas, cervical and thoracal vertebrae, while an insignificant trend could be observed in the ribs. FA fortification with 8 mg/kg prevented defects within the cervical vertebrae, with a trend toward prevention apparent in the true and false ribs. **(B–I)** In control animals, the arches of the cervical vertebrae only sporadically demonstrated an anomaly **(B)**, whereas irradiation lead to a notable presence of vertebral fusions (affecting two or three arches, shown by an arrow ← and arrowhead ◂, respectively) and excessive cartilage ≪ **(C)**. In controls, both the thoracic vertebrae and ribs never showed any anomalies **(D)**, but irradiated fetuses often lacked ribs (arrowhead ◂) and depicted excessive cartilage (double arrowhead ≪) and fusions (arrow ←) in the vertebrae **(E)**. In addition, the ribs also showed delayed ossification (arrowhead ◂) **(F)** and the vertebral bodies showed split ossification centers (arrowhead ◂) **(G)**. Finally, radiation also promoted the presence of a tilted sternum **(H,I)**. Data are represented as mean ± SEM, ^*^p ≤ 0.05, ^**^p ≤ 0.01, ^***^p ≤ 0.001, ^****^p ≤ 0.0001. Asterisks indicate a significant change as compared to the control FA + 1.0 Gy group of the respective skeletal region.

To assess general teratogenicity of X-ray exposure on the axial skeleton, alcian blue/alizarin red staining was utilized, a common methodology to assess the sub-macroscopic teratogenicity of chemical and physical agents (Young et al., 2000). X-irradiation at E7.5 increased the number of defects within the vertebrae, specifically in the atlas, the cervical vertebrae and the thoracal vertebrae when the animals were fed the control diet (Figure 4A, atlas, cervical and thoracal vertebrae). Within the cervical region, radiation primarily resulted in fused vertebrae and excessive cartilage (Figures 4B,C). At the thoracic level, the most common vertebral defects included fusions and excessive cartilage, whereas ribs were often missing (Figures 4D,E). Here, we also observed impaired ossification of the ribs (Figure 4F) and split ossification centers within the vertebrae (Figure 4G). Of note, irradiation also increased the incidence of a tilted sternum (Figures 4H,I). To a lesser extent, radiation lead to tilted vertebrae, displaced ribs, hooked (i.e., bent) ribs and short-length ribs (Supplementary Figures 1A–C). 8 mg/kg FA fortification prevented the occurrence of radiation-induced defects in the cervical region, whilst the 12 mg/kg diet group also showed a strong trend (henceforth defined as *P* = 0.05–0.08) toward prevention (Figure 4, cervical vertebrae). Surprisingly, a combination of 8 mg/kg FA and 1.0 Gy increased the number of defects within the caudal vertebrae, as compared to the 1.0 Gy irradiated animals that were fed the control diet (Figure 4A, caudal vertebrae). Furthermore, a trend was observed for the rescue of defects within the true and false ribs following fortification with the 8 mg/kg and 12 mg/kg diets (Figure 4A true and false ribs). Overall, FA fortification partially prevented skeletal defects within the cervical and thoracic vertebrae, whilst a trend toward prevention could be observed within the true and false ribs.

### Abnormal Adult Brain Morphology Following Pre-natal X-Ray Exposure

**Figure 5:**
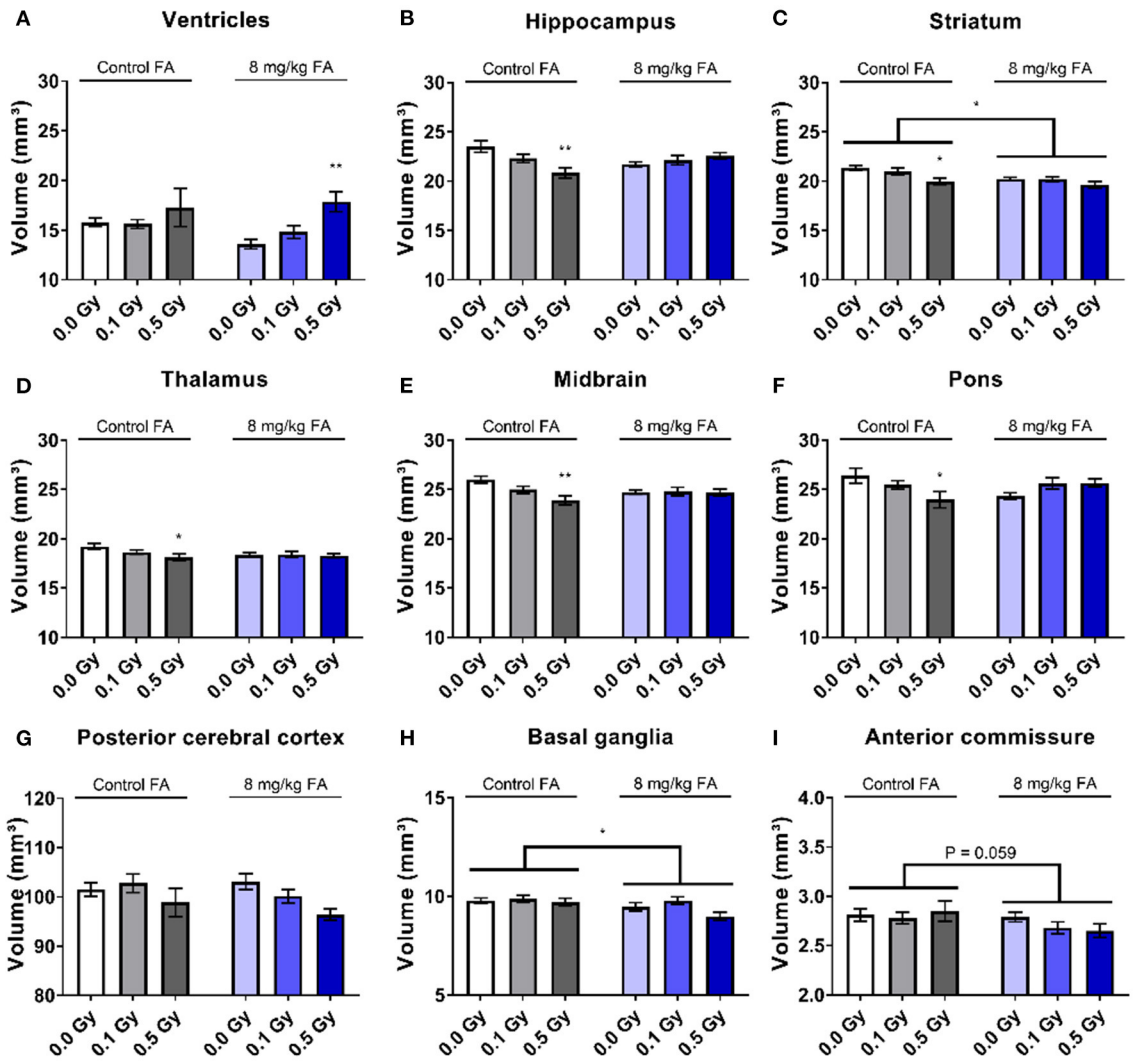
Volumetric analyses of various brain regions after pre-natal irradiation at E7.5. Ventricles were significantly increased after irradiation **(A)**, whereas the hippocampus **(B)**, striatum **(C)**, thalamus **(D)**, midbrain **(E)** and pons **(F)** were significantly smaller following a dose of 0.5 Gy in animals on the control diet. According to two way ANOVA, the radiation factor was significant in decreasing size of the posterior cerebral cortex **(G)**. FA by itself decreased the size of the basal ganglia **(H)** and the anterior commissure **(I)**. Data are represented as mean ± SEM, ^*^p ≤ 0.05, ^**^p ≤ 0.01.

We performed volumetric MRI analyses to assess whether the adult brain is structurally affected following irradiation at E7.5. Here we also assessed whether FA fortification could prevent any radiation-induced anomalies with inclusion of the 8 mg/kg FA diet, which was based on the rescue effect we observed in view of the radiation-induced fetal defects. We observed no differences in the volumes of whole brain, the olfactory system, the frontal cortex, the corpus callosum, the amygdala, the cerebellum and the corpora quadrigemina in response to radiation and/or FA (Supplementary Table 1). In contrast, other brain regions were affected by the radiation dose and the diet. Ventricles appeared significantly enlarged following irradiation [*F*_(2, 37)_ = 6.125; *P* = 0.0050] (Figure 5A), although no significance was reached when comparing individual radiation doses. We also found a radiation-induced reduction in volume of the hippocampus (Figure 5B), striatum (Figure 5C), thalamus (Figure 5D), midbrain (Figure 5E) and pons (Figure 5F) when the mothers were irradiated with 0.5 Gy. Of interest, an interaction effect between irradiation and the diet could be established for the hippocampus [*F*_(2, 37)_ = 4.654; *P* = 0.0157) (Figure 5B), midbrain [*F*_(2, 37)_ = 4.654; *P* = 0.0157] (Figure 5E) and the pons [*F*_(2, 37)_ = 3.792; *P* = 0.0318] (Figure 5F), which supports an FA-dependent rescue of radiation-induced size decrease. Furthermore, X-irradiation resulted in a trend toward a volumetric decrease of the posterior cerebral cortex [*F*_(2, 37)_ = 2.731; *P* = 0.0783] (Figure 5G) and the basal ganglia [*F*_(2, 37)_ = 2.768; *P* = 0.0758] (Figure 5H). Unexpectedly, FA food fortification reduced the size of the basal ganglia [*F*_(1, 37)_ = 4.961; *P* = 0.0321) (Figure 5H) and the striatum [*F*_(1, 37)_ = 7.067; *P* = 0.0115] (Figure 5C). A trend toward FA-induced size decrease was also observed for the anterior commissure [*F*_(1, 37)_ = 3.796; *P* = 0.0590] (Figure 5I).

### Irradiation Impairs Vision and Olfaction, Which Is Ameliorated by FA Fortification

**Figure 6:**
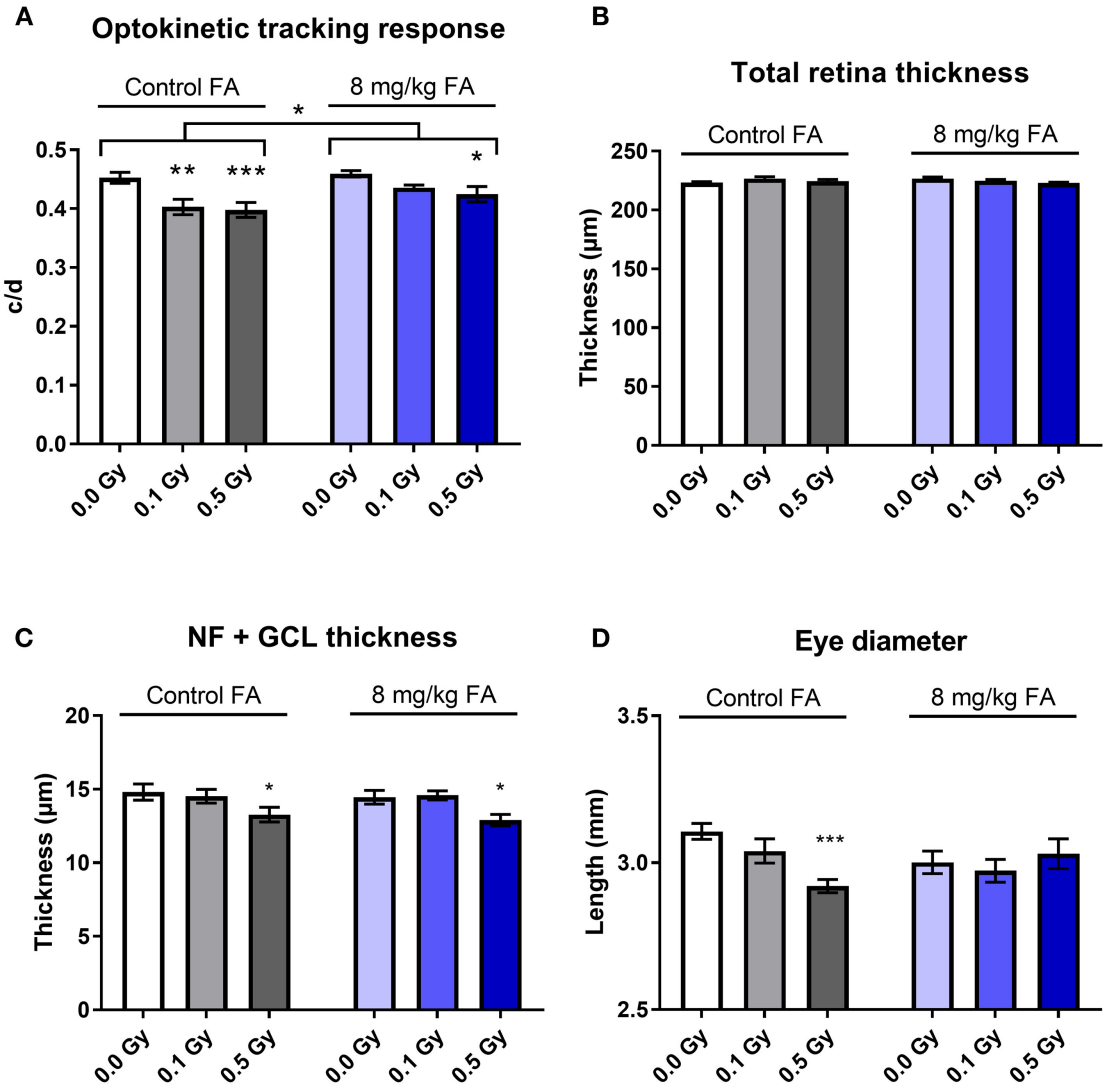
Visual acuity, retinal morphology, eye size and the impairment thereof by X-irradiation during neurulation. Radiation decreased visual acuity in adult mice (W5-W7) starting from 0.1 Gy, according to the optomotor test **(A)**. 8 mg/kg FA increased the irradiation dose required for a significant effect on optokinetic response to 0.5 Gy **(A)**. Irradiation or FA diet did not affect total retinal thickness, according to OCT analysis **(B)**, but a more detailed analysis showed that 0.5 Gy significantly reduced thickness of the nerve fiber + ganglion cell layer **(C)**. Using the MRI images for eye-size measurements, we identified a reduced axial length following 0.5 Gy, which was in turn ameliorated by FA fortification **(D)**. Data are represented as mean ± SEM, ^*^p ≤ 0.05, ^**^p ≤ 0.01, ^***^p ≤ 0.001.

In order to determine whether X-irradiation during neurulation can affect visual acuity later in life, and whether these effects could be countered by FA, a virtual optokinetic drum was used. Here, we observed that radiation decreased visual acuity [Figure 6A, *F*_(2, 64)_ = 10.02; *P* = 0.0002], whilst FA increased visual performance as compared to animals on the control diet [Figure 6A, *F*_(1, 64)_ = 6.565; *P* = 0.0128]. Furthermore, the impairment in acuity elicited by 0.1 Gy was alleviated by FA (Figure 6A). SD-OCT analysis did not show any changes in total retinal thickness following X-ray exposure or FA fortification (Figure 6B). Yet, a more detailed investigation revealed that the nerve fiber and retinal ganglionic cell layer (NF + GCL) thickness was decreased following 0.5 Gy irradiation [Figure 6C, *F*_(2, 63)_ = 7.618; *P* = 0.0011], which was not alleviated following the FA-rich diet. On the other hand, the high FA diet was shown to elicit a protective effect on the radiation-induced decrease in eye diameter (Figure 6D). Altogether, we showed a radiation-induced decrease in visual acuity starting from a low dose of 0.1 Gy onward, together with a reduced NF and GCL layer thickness and eye diameter following 0.5 Gy. FA prevented the decreased visual acuity elicited by 0.1 Gy, and rescued the 0.5 Gy-induced decrease in axial eye size.

**Figure 7:**
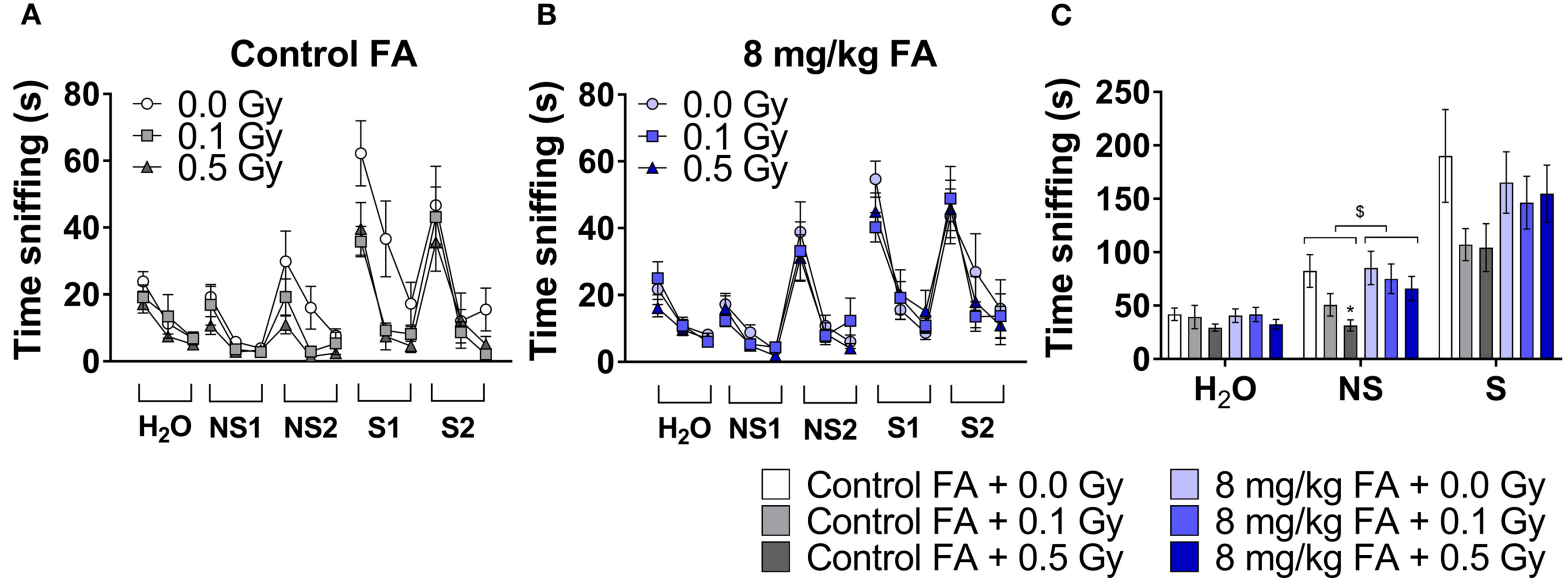
Odor habituation and dis-habituation in adult mice (W9–W11) following pre-natal irradiation at E7.5, under control or FA fortified diet. All animals show a typical approach behavior toward novel odors (dishabituation) and reduction in sniffing over time toward the same odor (habituation) **(A,B)**. Under control diet condition, irradiated animals show reduced sniffing time to novel odors **(A)**. In contrast, under high FA diet, sniffing time is at sham level **(B)**. The total amount of sniffing time toward a non-social odor, was reduced in irradiated animals under control diet, while high FA fortification increased the sniffing time to sham levels **(C)**. Data are represented as mean ± SEM, ^*^p ≤ 0.05 vs. sham (*post hoc*), $ *p* ≤ 0.05 control vs. FA diet (two-way ANOVA).

To investigate whether X-ray exposure during neurulation has an impact on olfactory performance and discrimination, and whether radiation-induced differences can be rescued by FA, we performed an olfaction-dependent habituation and dis-habituation test. When presented with a novel odor, mice will show specific approach and sniffing behavior (dishabituation, see Table 3), which increases further in the presence of S odors, and diminishes over the three time bins of 2 min (habituation). To compare the rate of habituation and dishabituation between the groups, we calculated the difference in sniffing time between (a) within the same odor of trial 1 and trial 3 (habituation) and (b) between odors from old to new odor (dishabituation). Habituation was observed in all conditions for all odors, indicating a normal loss of interest for odors over time (Figures 7A,B, Table 3). Similarly, approach behavior to a novel odor was observed in both sham and irradiated animals and was not affected by FA enrichment (Figures 7A,B, Table 3). However, irradiation reduced the total amount of time spent sniffing NS odors under control diet conditions compared to sham-irradiated animals (Figure 7C). Two-way ANOVA for factor diet (control diet or FA) and dose (sham, 0.1 and 0.5 Gy) during the NS odor presentation, indicated a significant effect for diet [*F*_(1, 63)_ = 4.078; *P* = 0.048] and for dose [*F*_(2, 63)_ = 3.908; *P* = 0.025] without significant interaction. *Post-hoc* analysis revealed a significant difference between sham- and 0.5 Gy-irradiation in the control diet group, indicating a reduced approach time to NS odors after irradiation. This reduced approach was alleviated when given the high FA diet. Of note, this reduced sniffing time is not due to an inability to approach, since presentation of S odors increased the sniffing time, but is possibly due to a decrease in attractiveness or detection of the odor itself. High FA diet normalized the sniffing time and approach to novel odors to baseline levels (Figures 7B,C), which is indicative of a protective role for FA. A similar trend was observed for S odors, however, the two way ANOVA did not indicate a significant effect of either factors. To conclude, irradiation in conjunction with the control diet resulted in hyposmia (i.e., a decreased sense of smell) for the NS odors, or a reduced interest in NS odors. These anomalies were alleviated when the diet was fortified with FA.

**Table 3:** Differences in sniff time used to assess habituation and dishabituation.

	**Control diet**			**8 mg/kg FA**		
Dose	0.0 Gy	0.1 Gy	0.5 Gy	0.0 Gy	0.1 Gy	0.5 Gy
**Habituation: difference in sniff time within same odor**						
Time (s)	−26.2 ± 3.2	−21.8 ± 3.7	−18.7 ± 2.1	−24.7 ± 2.2	−22.5 ± 3.2	−23.0 ± 3.3
**Dishabituation: difference in sniff time from old to new odor**						
Time (s)	30.6 ± 4.0	13.0 ± 3.9	20.4 ± 3.2	30.9 ± 4.1	25.3 ± 4.0	27.4 ± 3.2

*To quantify habituation (reduction in sniffing time to the same odor) and dishabituation (changes in sniffing time from old to new odor), we calculated the difference between t_x1_-t_x_ = Δt, and averaged the values across all odors for each animal. All Δt were different from 0 and there was no effect of radiation and/or FA. Data are represented as mean ± SEM*.

### No Changes in Activity and Motor Performance Following Irradiation of Animals on the Control Diet

**Figure 8:**
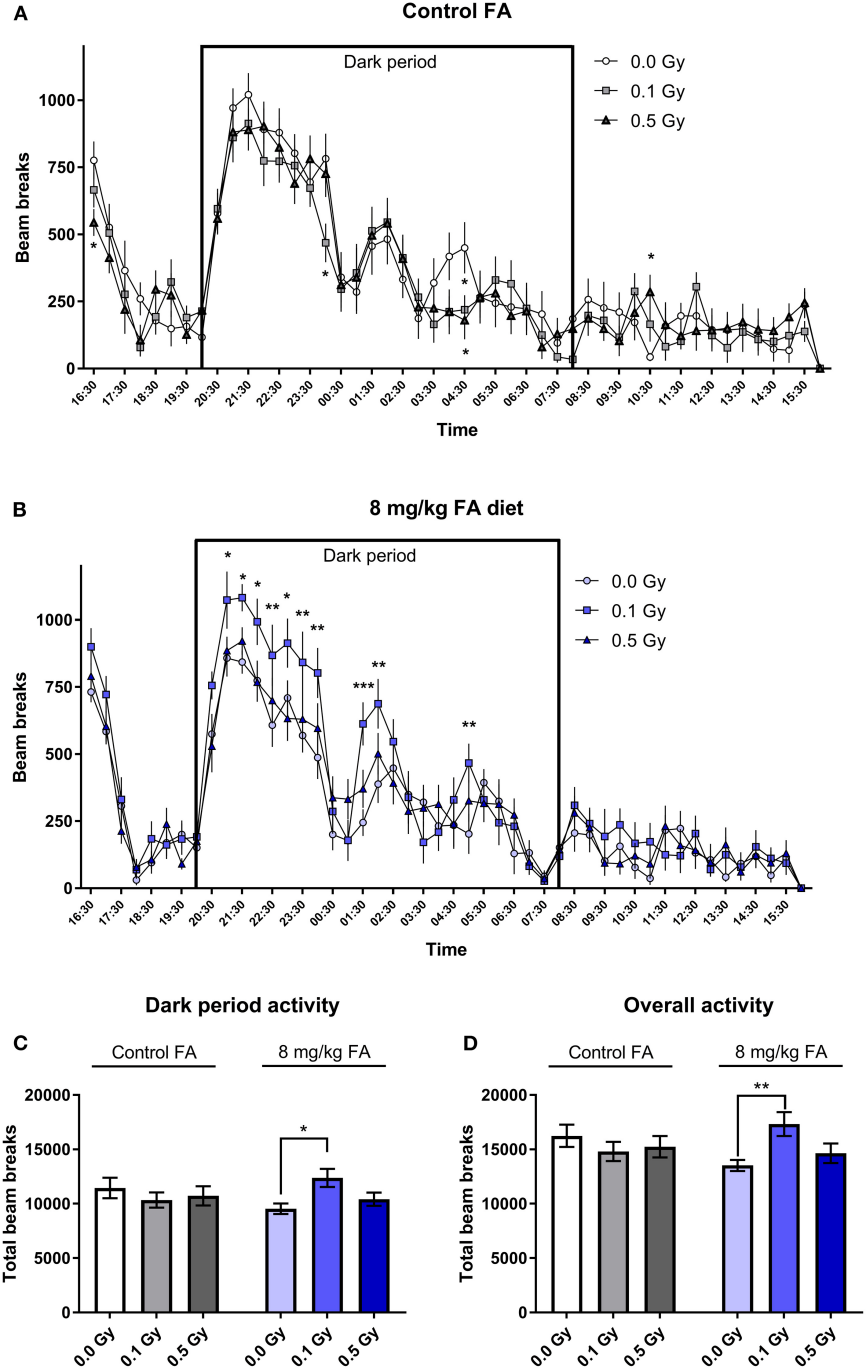
Cage activity in adult mice (W7–W9) and the impact of X-irradiation at E7.5 and FA food fortification. When the control diet was fed, no effect of pre-natal radiation exposure on cage activity was observed **(A)**. When animals were fed the FA diet, irradiation with 0.1 Gy at E7.5 significantly increased activity during the dark period, as tested in adult 7–9 week old mice **(B)**. **(C,D)** A summarized total of beam breaks during the dark period **(C)** and the overall experiment **(D)** confirmed the observations made in **(A,B)**. Data are represented as mean ± SEM, ^*^p ≤ 0.05, ^**^p ≤ 0.01, ^***^p ≤ 0.001.

General arousal and changes in circadian activity was assessed in the 23 h cage test. Under control diet conditions, radiation had no effect on the spontaneous activity, and all animals displayed the typical increase in night-time activity. Here, repeated measures (RM) ANOVA indicated a significant effect for time [*F*_(47, 1504)_ = 38.34; *P* < 0.0001], but not for radiation dose (Figure 8A). Animals on the high FA diet also showed a typical increase in night-time activity [*F*_(47, 1504)_ = 50.38; *P* < 0.0001). In addition, the high FA diet increased night-time activity in mice exposed to a low dose of 0.1 Gy [*F*_(2, 32)_ = 5.063; *P* = 0.0123) (Figures 8B,C). When animals were fed the high FA diet, repeated-measures (RM) ANOVA revealed a significant interaction effect between radiation and diet during the dark period [*F*_(2, 64)_ = 3.485; *P* = 0.0366], and *post-hoc* analysis indicated that only 0.1 Gy was significantly different from the sham-irradiated group (*P* = 0.0191). This interaction effect was also observed in the overall duration of the experiment [*F*_(2, 64)_ = 4.127; *P* = 0.0206] (Figure 8D).

Balance and coordination was tested on the accelerating rotarod, but radiation had no effect on motor coordination, and we also saw no effect of FA diet (Supplementary Figure 2).

### Radiation Did Not Affect Overall Cognition, but FA Adversely Altered Social Behavior

#### Open Field and Social Exploration

**Figure 9:**
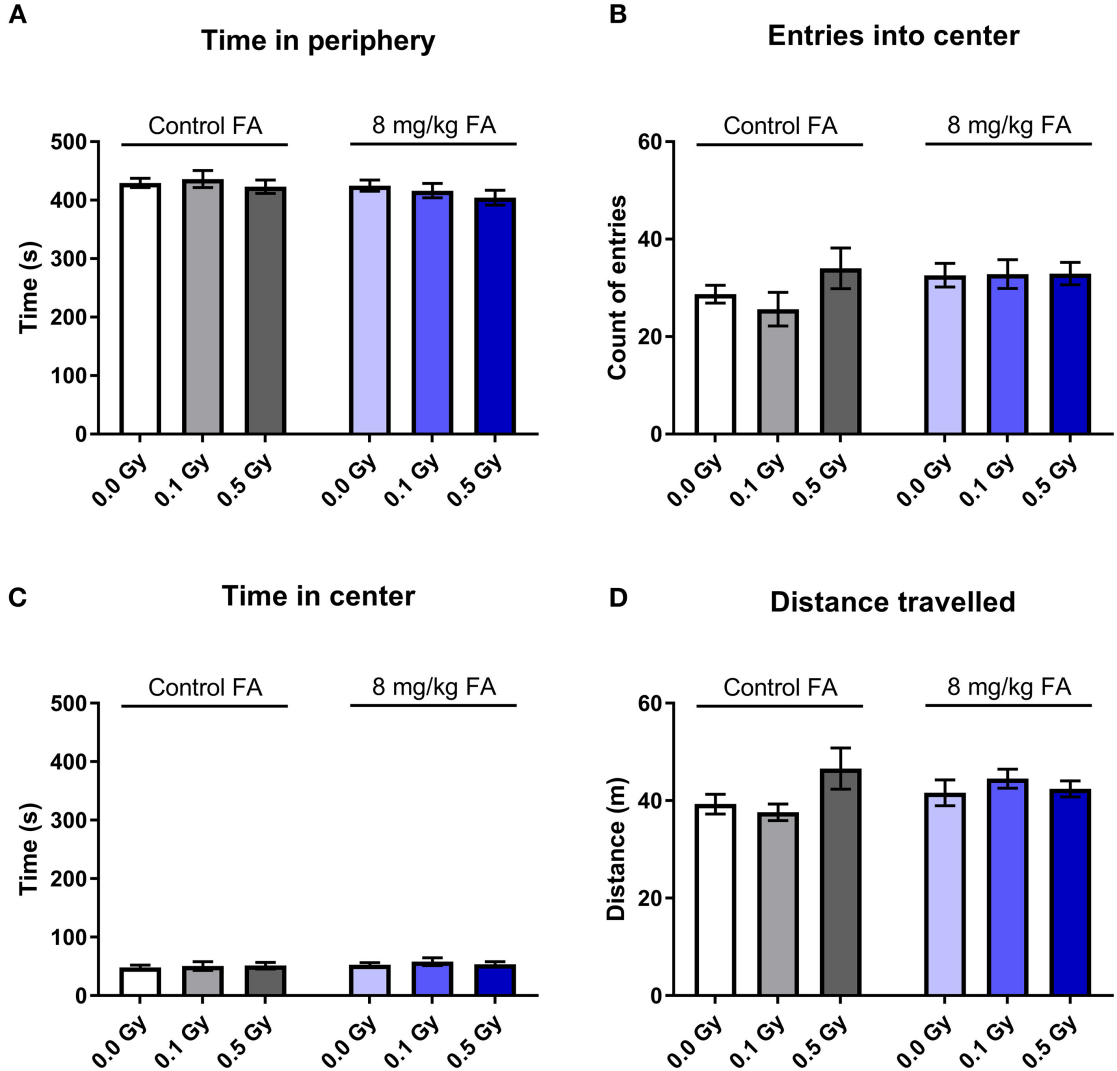
Effect of embryonic X-irradiation and FA fortification on exploration behavior in adult mice, according to the open field test. Neither E7.5 irradiation, nor diet had an impact on the time spent in the periphery **(A)**, entries into the center **(B)**, time spent in the center **(C)** and the total distance traveled **(D)**. Data are represented as mean ± SEM.

**Figure 10:**
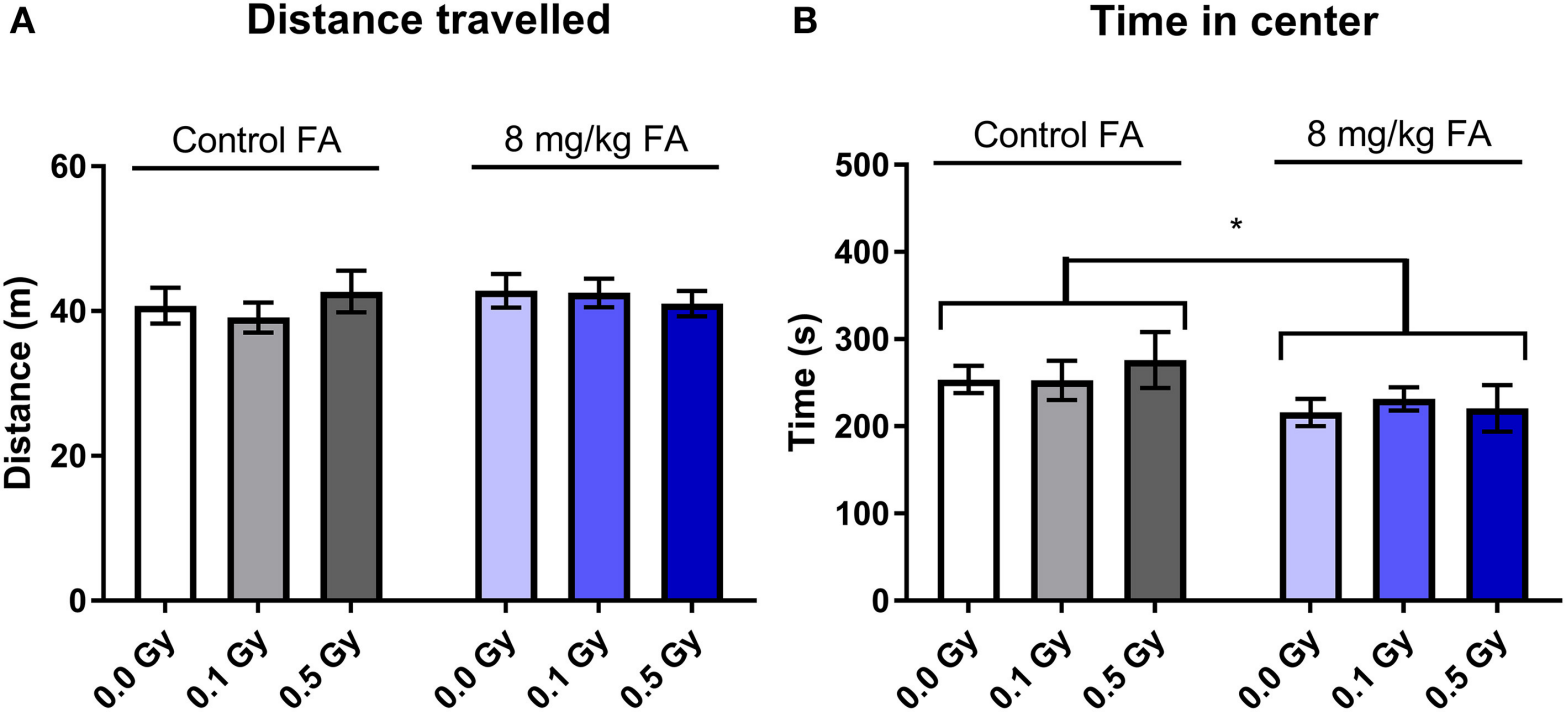
Social exploration in an adapted open field test was unaffected by 1.0 Gy exposure at E7.5, but was impaired by FA fortification. Neither radiation, nor FA diet had an impact on exploration behavior in adult mice, according to the total distance traveled in the arena **(A)**. In contrast, FA fortification resulted in animals spending less time in the center, in close proximity to the unknown mouse **(B)**. Data are represented as mean ± SEM, ^*^p ≤ 0.05.

The open field test was used to assess exploratory behavior in a novel and stressful environment. The animals are dark-adapted and then placed in a brightly illuminated open field for 10 min. Anxious animals will spend their time close to the walls and will not enter the open center zone. We observed that all groups spent most of their time close to the wall, and there was no effect of radiation nor of FA enriched diet on the time spent in the periphery (Figure 9A). Center visits were frequent (Figure 9B) but overall rather short (Figure 9C). Furthermore, the distance the animals traveled over 10 min was similar in all groups (Figure 9D). Likewise, other parameters, such as latency to enter the center, walking speed or distance traveled in the center were also not different between the groups (respectively, Supplementary Figures 3A–C). We used a modified setup to evaluate social approach: two stranger mice were placed in the center of the arena and provide an attraction point for the test mouse. We observed similar distance covered in all groups (Figure 10A), but FA fortification decreased the time spent in the center as compared to animals on the control diet [Figure 10B, *F*_(1, 63)_ = 4.316; *P* = 0.0418]. Factors such as center distance, center entries, mean speed, time in periphery and latency to enter the center were unaltered (respectively, Supplementary Figures 4A–E).

#### Elevated Plus Maze

**Figure 11:**
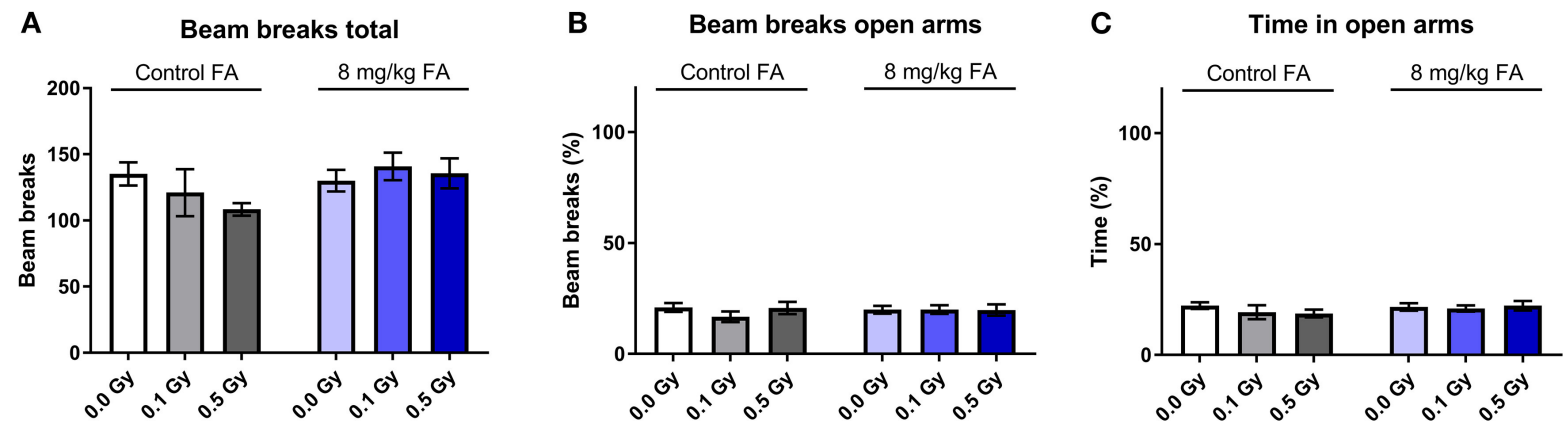
Effect of embryonic X-irradiation and FA fortification on anxiety, tested in the elevated plus maze. Neither irradiation at E7.5 nor diet had an effect on the total number of beam breaks **(A)**, beam breaks in the open arms **(B)** or time spent in the open arms **(C)** by adult animals. Data are represented as mean ± SEM.

The EPM is considered the typical test to assess anxiety related exploration. We observed no significant difference in total beam breaks between the different groups. Two-way ANOVA indicated no effect of factor radiation [*F*_(2, 63)_ = 0.4912; *P* = 0.6142] nor of diet [*F*_(1, 63)_ = 2.206; *P* = 0.1424) on total beam breaks (Figure 11A). Open arm visits (Figure 11B) and open arm dwell (Figure 11C), both readouts for anxiety, were similar in all groups. In general, neither radiation exposure nor diet had an impact on anxiety-related activity.

#### Morris Water Maze

**Figure 12:**
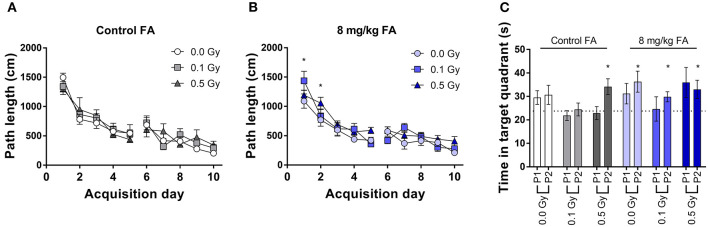
Spatial place learning and reference memory of adult mice in the MWM, following X-ray exposure at E7.5 and/or FA food fortification (8 mg/kg FA). All groups learned to located the hidden platform under control diet **(A)** and FA fortification **(B)**. Reference memory was assessed during interspersed probe trials **(C)**. In FA enriched diet, all animals displayed significant preference to the target quadrant above chance level (25%). Data are represented as mean ± SEM, ^*^p ≤ 0.05 to chance level (25 %).

During place learning, under control diet, all groups learned in a similar way to locate the hidden platform (Figure 12A). RM ANOVA indicated an effect for day [*F*_(9, 270)_ = 41.4; *P* < 0.001], but not for radiation dose and no interaction. In contrast, under FA conditions, RM ANOVA indicated an effect for day [*F*_(9, 288)_ = 37.0; *P* < 0.001], and an effect of dose [*F*_(2, 288)_ = 4.34; *P* = 0.022), without interaction (Figure 12B). *Post-hoc* analysis indicated, as compared to sham-irradiated animals, a significantly longer path length in 0.5 Gy-irradiated animals only on the first 2 days [*q*_(3)_ = 4.0; *P* = 0.013, and *q*_(3)_ = 3.6; *P* = 0.029] (Figure 12B). However, this finding was considered biologically irrelevant due to the low number of days affected. The probe trials were interspersed after day 5 and 10. Target quadrant preference was evaluated by comparing time spent in the target quadrant with chance level (25%). Under control diet conditions, 0.1 Gy-irradiated mice showed a clear lack of target quadrant preference even after 2 weeks of training, which was also true for 0.5 Gy-irradiated animals during the first probe trial (Figure 12C). When the diet was FA fortified, all radiation-dose groups demonstrated significant target quadrant preference during the second trial (Figure 12C), suggestive for a FA-induced amelioration of reference memory and supporting FA to have a role in learning and memory. Nonetheless, these results are to be interpreted with caution due to the relatively low numbers of animals being included in the analysis.

#### Passive Avoidance

The effect of pre-natal X-ray exposure and FA on amygdala and hippocampal dependent fear-related memory formation was tested using the passive avoidance set-up. Animals on the control diet [*F*_(1, 30)_ = 86.87; *P* < 0.0001] and on the high FA diet [*F*_(1, 30)_ = 219.7; *P* < 0.0001] demonstrated an increased latency to enter the dark chamber after the shock (Supplementary Figure 5A). A comparison between animals on the control diet and on the high FA diet revealed no interaction between diet and latency [Supplementary Figure 5B, *F*_(1, 18)_ = 0.1248; *P* = 0.7280]. These data suggest that neither radiation nor high FA diet has an impact on passive avoidance learning.

## Discussion and Conclusion

### Radiation-Induced Anophthalmos, Exencephaly and Gastroschisis Are Prevented by FA

X-irradiation at E7.5 induced various congenital eye defects, exencephaly, agnathia and gastroschisis in the offspring. Interestingly, the right eye appeared more susceptible toward radiation-induced anophthalmos as compared to the left eye. This observation is in line with our previous study (Craenen et al., 2017) and could be explained by the used mouse strain with a C57BL6/J genetic background. C57BL6/J mice have a strong natural tendency toward developing asymmetrical eye defects, with a bias toward right-eye anophthalmos/microphthalmos (Smith et al., 1994). Alternatively, in the developing embryo there are various gestational stages that demonstrate left-right asymmetry (e.g., various signaling mechanisms). Even the developing eye is known to exhibit such developmental asymmetry (Levin, 2005), hypothetically allowing potential teratogens such as ionizing radiation to interfere with the left-right axis during embryogenesis. Hence, to assess why radiation induces an asymmetric eye phenotype, it is of interest to compare our results to a different mouse strain, and to more closely explore the molecular mechanisms along the embryonic left-right axis after X-irradiation.

In this study, we demonstrated for the first time that FA fortification (8 mg/kg FA and 12 mg/kg FA) prevents radiation-induced anophthalmos, exencephaly and agnathia. Already in the 1960's a link between FA intake and the incidence of congenital EDs was suggested. For instance, maternal FA-deficiency was shown to increase the risk of EDs in rats (Armstrong and Monie, 1966). A later study supported these findings, where a FA-deficient diet in mice could lead to anomalies such as anophthalmos and microphthalmos (Maestro-de-las-Casas et al., 2013). Furthermore, ethanol-induced retinal anomalies were rescued with FA supplementation in zebrafish (Muralidharan et al., 2015). In contrast, an epidemiological study could not determine a link between FA intake and the risk for anophthalmos and microphthalmos. Yet, the authors conceded that several caveats such as a small case population and the lack of clinical analyses of key biomarkers may have impaired proper effect estimation (Shaw et al., 2007). The second category of radiation-induced birth defects that appears folate-responsive, is exencephaly. The prevention of exencephaly by FA is well-described in literature, albeit not with ionizing radiation as the effecting teratogen, but with e.g., ethanol (Yanaguita et al., 2007) and glucose (Wentzel and Eriksson, 2005; Oyama et al., 2009). Of note, international FA food fortification initiatives have already successfully decreased the incidence of NTDs (Blom et al., 2006). The third category of radiation-induced birth defects, that is partially preventable by FA, is agnathia. In humans, agnathia is a very rare congenital disorder, commonly classified within the otocephaly family of disorders (incidence <1/70 000 births) (Gekas et al., 2010; Herman et al., 2012; Jagtap et al., 2015; Sergouniotis et al., 2015). To our knowledge, the only published observation where FA fortification could prevent agnathia was in *Twisted gastrulation* mutant mice, which have a high penetrance of midline facial defects and jaw defects (Billington et al., 2013). Even though our study is the first to demonstrate the prevention of X-ray-induced anophthalmos, exencephaly, and agnathia, the rescue is only partial. It would be of interest to further explore the efficacy of other radioprotectant compounds, potentially in combination with FA, in preventing these defects.

In contrast to the defects discussed above, we observed no folate-responsiveness of radiation-induced iris anomalies, open eye and gastroschisis. The iris anomaly observed in our study was characterized by a strongly decreased pupil size, with the most severe cases having no apparent pupil at all (Smith et al., 1994; Craenen et al., 2017). In accordance, in a previous study on hyperthermia-induced iris anomalies, no protective effect of FA could be found (Czeizel et al., 2011). Yet, there are to our knowledge no other publications that have previously investigated the efficacy of FA in preventing open eye anomalies. Hereto, it might be worthwhile to investigate the protective effect of thyroxine supplementation on radiation-induced open eyes, as some success was already made with this hormone (Juriloff, 1985). With regard to gastroschisis, it remains severely debated whether these defects can be prevented with FA, with efficacy strongly depending upon the acting teratogen (Godwin et al., 2008; Paranjothy et al., 2012; Yang et al., 2016).

### Reduced Fetal Weight and Increased Pre-natal Death Following Irradiation Are Ameliorated by FA

Aside from gross macroscopic defects, other aspects of pre-natal development were also assessed. Irradiation significantly reduced fetal weight at E18, as was also observed in a previous study (Craenen et al., 2017), which was not notably ameliorated by FA. This stands in contrast to the teratogen ethanol, where embryotoxic weight loss can be prevented by FA (Xu et al., 2006). However, a potentially adverse outcome observed after fortification of the highest dose of FA was the increase of fetal weight at E18. Epidemiological studies already reported that increased fetal weight is a consequence of FA fortification (Tamura and Picciano, 2006; Balarajan et al., 2013; Li et al., 2016; Ramakrishnan et al., 2016), which is linked to type 2 diabetes and adult obesity (Curhan et al., 1996; Johnsson et al., 2015). Both the number of late fetal deaths and resorptions were increased following irradiation, which is consistent with previous work (Pampfer and Streffer, 1988; Kim et al., 2001; Craenen et al., 2017). These cases of pre-natal mortality were reduced by FA fortification, which is in line with epidemiological studies that focused on fetal loss (Andersen et al., 2010) and miscarriage (Byrne, 2011).

### Radiation-Induced Fetal Morphological Defects, and Prevention With FA

Since the mid-twentieth century, it has already been known that exposure to (high) doses of ionizing radiation during pregnancy can result in a variety of axial and appendicular skeletal defects (Jarmonenko, 1988). These studies focused mostly on severe spinal defects such as spina bifida, which are known to result from exposure to external radiation sources such as neutrons and γ-rays (A-bombs) and internal contamination from e.g., depleted uranium (commonly used in munitions) [reviewed by Hindin et al. (2005)]. In our study, pre-natal irradiation had the most detrimental impact on the cervical and thoracic vertebrae. This was also observed in a study by Russell, who used 2.0 Gy of X-rays at E7.5 (Russell, 1956). A contrasting study described more malformations in the ribs of CRI mice than in the vertebrae, following 2.0 Gy-γ-irradiation at E7.5 (Kim et al., 2001). This difference might be mouse strain dependent, or might result from variations in radiation dose and type. In further support of our study, a dose-dependent induction of skeletal malformations after irradiation (0.5 Gy to 4.0 Gy) at E11.5 was observed (Kim et al., 2001). It would therefore be interesting to further investigate this dose-dependency and the existence of a dose-threshold in our experimental set-up. Intriguing was the presence of split spinal ossification centers after irradiation, which could lead to open vertebral arches and potentially spina bifida occulta in later life (Regnier et al., 2002). Altogether, we are the first to explore in such detail developmental defects in the axial skeleton after irradiation at E7.5, and to demonstrate that FA fortification can significantly reduce the risk for radiation-induced skeletal defects.

Although we observed an increase in axial skeletal defects after 1.0 Gy irradiation, it appears that the sub-lethal doses (Craenen et al., 2017) used for the behavioral assays (≤ 0.5 Gy) might have been too low to elicit any functional detriment. Indeed, in terms of motor performance, none of the behavioral tests could identify a clear impairment following irradiation, as is discussed in more detail below.

### Persistent Radiation-Induced Defects in the Adult Nervous System and the Preventive Role of FA

Because pre-natal exposure to ionizing radiation can induce gross congenital central nervous system defects (e.g., microphthalmos and anophthalmos) at moderate to high X-ray doses (0.5–1.0 Gy) (Craenen et al., 2017), we decided to explore whether this can elicit functional and morphological neurological defects that persist into adult age.

We used *in vivo* MRI to investigate whether X-ray exposure during neurulation has an effect on adult brain and eye morphology. Although we observed no global microcephaly, as was shown after irradiation during neurogenesis (Verreet et al., 2015, 2016a), volumetric analyses unveiled a decreased volume of some dedicated brain areas. More specifically, we observed that 0.5 Gy significantly reduces the size of the hippocampus, striatum, thalamus, midbrain and pons. These structures are involved in various mechanisms, ranging from cognition to visual acuity. For example, the thalamus is known for its importance in processing and relaying visual information (Tyll et al., 2011). The pons and midbrain are also involved in visual functioning, as anomalies within these brainstem regions can result in both horizontal and vertical gaze palsy (Strupp et al., 2014; Lin et al., 2018). Furthermore, we observed that irradiation with 0.5 Gy decreased the axial length of the adult eye, which can be relayed directly to an increased incidence of radiation-induced microphthalmia (Verma and Fitzpatrick, 2007; Craenen et al., 2017), and can be associated to an increased risk of refractive errors (Bhardwaj and Rajeshbhai, 2013). Supporting the MRI-based findings, SD-OCT revealed a decreased thickness of the NF+GCL layer in the adult eye, following 0.5 Gy at E7.5, which might lead to a decreased visual acuity (Moster et al., 2016).

Concomitant with these radiation-induced alterations in the brain and the observed eye anomalies, we indeed observed a decreased visual acuity following E7.5 irradiation. Interestingly, this was also observed in 0.1 Gy-irradiated animals, that did not show a decreased eye size and NF+GCL thickness nor a reduction in brain volumes, suggesting that other mechanisms might also be involved. The morphological defects underlying the radiation-induced loss of visual acuity may thus extend beyond changes in eye structure and warrants further investigation.

Even though pre-natal irradiation had no marked impact on the olfactory system in the adult brain, behavioral tests for olfaction were included in the test battery. This decision was based on a previous study that showed transient transcriptional disturbances in the embryonic head following 1.0 Gy irradiation at E7.5 that were related to the development of the olfactory epithelium (Craenen et al., 2020b). This is an important observation, as the olfactory system starts to develop during this neurulation period (Treloar et al., 2010). Besides, the overall process of olfaction extends well-beyond the olfactory lobe (Lehmkuhl et al., 2014), with congenital anomalies within the peripheral olfactory system (e.g., the olfactory epithelium) having been linked to hyposmia (Bergman et al., 2010). We are the first to demonstrate a decreased olfactory acuity in adult mice following 0.5 Gy at E7.5. In particular, we could demonstrate a more pronounced anosmia for NS than S odors, which could be attributed to the functional importance of social odors and/or chemical differences between the respective odorants (Sinding et al., 2017). Since defects at this level might explain the observed hyposmia, it is of interest for future studies to more closely investigate this complex structure following pre-natal X-irradiation.

In contrast to the morphological and sensory anomalies discussed above, we observed no effect of irradiation on cognition. Previous behavioral screenings demonstrated that irradiation of mice during neurogenesis had a marked effect on memory-based performance (Verreet et al., 2015, 2016a). However, when we irradiated animals during neurulation, no evidence for impaired memory formation or retention could be observed, both for spatial and fear-dependent learning. The observation that 0.5 Gy irradiation decreases hippocampal volume may appear paradoxical in that sense, but this volumetric decrease might not be directly related to a loss in function. Although irradiated animals had a notable loss of visual acuity, the defect may have been too small to influence MWM performance, which depends on large and distinct visual cues (Lindner et al., 1997; Brown and Wong, 2007; Phillips et al., 2013; Vorhees and Williams, 2014).

Many of the radiation-induced anomalies that were observed in adult mice could be (in part) prevented by FA fortification. FA fortification by itself had only a minimal impact on the adult tests. For instance, we observed a decreased social exploration in mice on the FA-fortified diet and also identified a lower volume of the basal ganglia (i.e., caudate putamen and adjacent structures). This might be related to the social impairments in these mice, since a decreased basal ganglia volume was already linked with autism-spectrum disorder (Barua et al., 2014), but whether FA fortification is a risk factor for abnormal social behavior remains controversial [reviewed in Wiens and DeSoto (2017)]. Furthermore, this volumetric loss might have played a role in the changes in cage activity as well, because the basal ganglia are involved in the regulation of the sleep-wake cycle (Qiu et al., 2010) and general activity (Portmann et al., 2014).

It is important to note that, depending on the causative factor, congenital defects may respond in a dose-dependent manner to FA fortification, with higher doses of FA yielding lower defect prevalences (Gray and Ross, 2009). Yet, in our study, the radioprotective role of FA did not appear dose-responsive and no added benefit was noted following 12 mg/kg FA fortification. Hence, we decided to limit the studies in adult animals to the 8 mg/kg FA diet. Radiation-induced volumetric decreases of the hippocampus, striatum, thalamus, midbrain and pons were prevented with FA fortification. In addition, FA rescued visual acuity loss following a dose of 0.1 Gy, but not 0.5 Gy. Yet, not all morphological eye anomalies were prevented by FA, for instance the radiation-induced reduction of NF+GCL thickness. Finally, radiation-induced hyposmia for NS odors was alleviated by AF. To the best of our knowledge, were are the first to highlight this radioprotective/antiteratogenic character of FA. In all, we can conclude that X-ray exposure during neurulation affects the adult nervous system at both a morphological and functional level, from a dose of 0.1 Gy onward, and that these defects can be in part prevented by FA food fortification. In the context of radiation protection, our study supports the use of FA fortification to increase the dose threshold required to elicit adult brain and eye anomalies.

### Potential Mechanisms Underlying FA-Mediated Radioprotection

Although the exact mechanism through which FA elicits its radioprotective role is currently unknown, it is still of interest to highlight several likely modes of action. A first hallmark consequence of ionizing radiation exposure is the generation of reactive oxygen and nitrogen species, which can in turn damage various cellular structures. The detrimental impact of excessive oxidative stress in the developing embryo and pregnant mother has been repeatedly addressed in literature. Indeed, it appears that a disturbed redox status is a recurrent theme in the etiology of birth-defects caused by various chemicals, including thalidomide, phenytoin and ethanol (Dennery, 2007). FA is known to have antioxidative properties *in vitro*, which is suggestive of its potential radioprotective effect, but it remains unclear whether this antioxidative role persists at a systemic level *in vivo*. A second hallmark consequence of irradiation is DNA-damage (Reisz et al., 2014), which can theoretically be repaired more efficiently with an increased access to one-carbon donors such as FA. A third hallmark consequence of irradiation includes epigenetic alterations, in particular DNA methylation. Folates fulfill an important role in DNA methylation (Crider et al., 2012). As the key one-carbon donor behind the methylation process, it stands to reason that changes in the folate pool would affect this epigenetic process and potentially reverse radiation-induced DNA hypomethylation. The fourth potential mode-of-action lies in radiation-induced changes in the transcriptome and proteome. We previously demonstrated that X-irradiation (1.0 Gy) at E7.5 in mice reduced the expression of Lhx2, a key transcription factor for eye, brain and olfactory development (Craenen et al., 2020b). Furthermore, mutations in genes associated with Lhx2 are known to cause birth defects such as exencephaly (Barbera et al., 2002). As such, it is of interest to assess whether the radiation-induced suppression of Lhx2 transcription/translation can be alleviated by FA fortification. Although this study does not address any of the aforementioned modes-of-action directly, an exploration of the mechanisms that might be involved in the antiteratogenic and radioprotective effect of folic acid is warranted. Such novel insights might contribute to developing even more efficient means to protect the unborn child from genotoxic hazards such as radiation.

## Conclusion

FA food fortification is effective at partially preventing the embryotoxic effects of X-ray exposure. Specifically severe defects such as anophthalmos, exencephaly and agnathia were responsive to FA. In addition, late fetal deaths, the incidence of resorptions, fetal weight and skeletal defects within the cervical and thoracal vertebrae were all negatively affected by 1.0 Gy X-irradiation at E7.5, which was in turn partially countered by FA. Behavioral studies demonstrated that X-ray exposure to sub-lethal doses (≤ 0.5 Gy) at E7.5 resulted in a decrease of visual acuity and olfactory performance in the habituation/dishabituation test. The impaired visual performance was supported by radiation-induced loss of NF+GCL thickness and a decreased eye diameter, at least for the highest dose of 0.5 Gy. We can conclude from our MRI data that irradiation during neurulation has more site-specific consequences than irradiation during neurogenesis (Verreet et al., 2015, 2016a). As such, it would be of interest to follow up this study with more sensitive behavioral tests, tailored more specifically to those brain regions that are decreased in volume following X-irradiation.

The increasing exposure of humans to ionizing radiation is a contemporary topic that deserves proper investigation. The heightened exposure to ionizing radiation finds its roots in the clinical environment, nuclear disasters, war or terrorist activities and natural sources such as Radon gas. With this research paper, the authors wish to address and promote novel radioprotection strategies such as FA fortification and the future implementation thereof in high-risk groups that currently do not have access to FA-fortified staple foods (or FA supplements). Included in these risk-groups are e.g., pregnant patients who require radiodiagnostics or radiotherapy and pregnant women living in radioisotope-contaminated regions. The fetal doses that can be expected during clinical exposure events (including conventional radiotherapy, computed tomography and nuclear medicine) range from 0.01 to 43.9 mGy (Lazarus et al., 2009). These doses are lower than those used in this study, as we opted to reduce the number of animals required to observe significant radiation effects. As such, it is difficult to make a direct extrapolation from the animal research presented here to the human exposure scenarios listed above. Nonetheless, as a proof of concept this study demonstrates the potential for using FA fortification to protect the unborn child against ionizing radiation. Protecting the unborn child from the detrimental effects of ionizing radiation will improve their quality of life, by preventing radiation-induced birth defects and sensory deprivation. Although our study in mice indicates that *ad libitum* FA food fortification at 8 mg/kg is sufficient to provide a radioprotective effect, the optimal concentration for humans remains to be studied in the context of radiation protection. Considering both the promising results and the limitations of this study, the authors support larger (epidemiological) studies (with lower fetal radiation doses) to explore the use of FA as a radioprotectant in humans.

## Data Availability Statement

The raw data supporting the conclusions of this article will be made available by the authors, without undue reservation.

## Ethics Statement

The animal study was reviewed and approved by Institutional Ethical Committees of SCK-CEN/VITO (ref. 02–012) and the Animal Welfare Committee of the KU Leuven.

## Author Contributions

MB and MV mentored KC, LM supervised KC as University promotor. KC, MV, LM, and MB planned the experiments and offered guidance regarding experimental design. KC performed the majority of the lab work and wrote the manuscript. LC, JB, and MN contributed significantly to lab work. KG processed the MRI images and WG assisted with the MRI image capture procedures. ZC-V and RD'H offered invaluable guidance and assistance regarding the behavioral tests. MB, MV, LM, KG, SB, ZC-V, RD'H, and UH reviewed the initial manuscript and offered crucial feedback that contributed to the submitted paper. KC was a joint PhD student of SCK CEN and KU Leuven funded through a scholarship of SCK CEN. All authors contributed to the article and approved the submitted version.

## Conflict of Interest

The authors declare that the research was conducted in the absence of any commercial or financial relationships that could be construed as a potential conflict of interest.

## Abbreviations

EPM: Elevated plus maze
E: embryonic day
ED: eye defect
FA: folic acid
MRI: magnetic resonance imaging
MWM: Morris water maze
NF+GCL: nerve fiber + retinal ganglionic cell layer
NTD: neural tube defect
NS: non-social odor
RARE: rapid acquisition relaxation enhancement
RM: repeated measures
S: social odor
SD-OCT: spectral domain optical coherence tomography.

## References

[B1] Andersen G. S. Friis H. Michaelsen K. F. Rodrigues A. Benn C. S. Aaby P.. (2010). Effects of maternal micronutrient supplementation on fetal loss and under-2-years child mortality: long-term follow-up of a randomised controlled trial from Guinea-Bissau. Afr. J. Reprod. Health 14, 17–26. Available online at: http://www.ncbi.nlm.nih.gov/pubmed/21243915 (accessed January 5, 2018). 21243915

[B2] Arbuckle E. P. Smith G. D. Gomez M. C. Lugo J. N. (2015). Testing for odor discrimination and habituation in mice. J. Vis. Exp. 99:e52615. 10.3791/52615 25992586 PMC4542325

[B3] Armstrong R. C. Monie I. W. (1966). Congenital eye defects on rats following maternal folic-acid deficiency during pregnancy. J. Embryol. Exp. Morphol. 16, 531–42. Available online at: http://www.ncbi.nlm.nih.gov/pubmed/4960285 (accessed August 22, 2014). 4960285

[B4] Balarajan Y. Subramanian S. V. Fawzi W. W. (2013). Maternal iron and folic acid supplementation is associated with lower risk of low birth weight in India. J. Nutr. 143, 1309–1315. 10.3945/jn.112.172015 23761647

[B5] Barbera J. P. M. Rodriguez T. A. Greene N. D. E. Weninger W. J. Simeone A. Copp A. J.. (2002). Folic acid prevents exencephaly in Cited2 deficient mice. Hum. Mol. Genet. 11, 283–93. 10.1093/hmg/11.3.283 11823447

[B6] Barua S. Chadman K. K. Kuizon S. Buenaventura D. Stapley N. W. Ruocco F.. (2014). Increasing maternal or post-weaning folic acid alters gene expression and moderately changes behavior in the offspring. PLoS ONE 9:e101674. 10.1371/journal.pone.0101674 25006883 PMC4090150

[B7] Bergman J. E. H. Bosman E. A. van Ravenswaaij-Arts C. M. A. Steel K. P. (2010). Study of smell and reproductive organs in a mouse model for CHARGE syndrome. Eur. J. Hum. Genet. 18, 171–177. 10.1038/ejhg.2009.158 19809474 PMC2987182

[B8] Bhardwaj V. Rajeshbhai G. P. (2013). Axial length, anterior chamber depth-a study in different age groups and refractive errors. J. Clin. Diagn. Res. 7, 2211–2212. 10.7860/JCDR/2013/7015.3473 24298478 PMC3843406

[B9] Billington C. J. Schmidt B. Zhang L. Hodges J. S. Georgieff M. K. Schotta G.. (2013). Maternal diet supplementation with methyl donors and increased parity affect the incidence of craniofacial defects in the offspring of twisted gastrulation mutant mice. J. Nutr. 143, 332–339. 10.3945/jn.112.168906 23343680 PMC3713022

[B10] Blom H. J. Shaw G. M. den Heijer M. Finnell R. H. (2006). Neural tube defects and folate: case far from closed. Nat. Rev. Neurosci 7, 724–731. 10.1038/nrn1986 16924261 PMC2970514

[B11] Bollen B. Ramanantsoa N. Naert A. Matrot B. Van den Bergh O. D'Hooge R.. (2015). Emotional disorders in adult mice heterozygous for the transcription factor Phox2b. Physiol. Behav. 141, 120–126. 10.1016/j.physbeh.2015.01.012 25582512

[B12] Brown R. E. Wong A. A. (2007). The influence of visual ability on learning and memory performance in 13 strains of mice. Learn. Mem. 14, 134–144. 10.1101/lm.473907 17351136 PMC1838554

[B13] Burrow G. N. Hamilton H. B. Hrubec Z. Amamoto K. Matsunaga F. Brill A. B. (1964). Study of adolescents exposed in utero to the atomic bomb, Nagasaki, Japan. I. General aspects: clinical and laboratory data. Yale J. Biol. Med. 36, 430–44. Available online at: http://www.pubmedcentral.nih.gov/articlerender.fcgi?artid=2604646andtool=pmcentrezandrendertype=abstract (accessed August 9, 2014). 14173443 PMC2604646

[B14] Byrne J. (2011). Periconceptional folic acid prevents miscarriage in Irish families with neural tube defects. Ir J Med Sci. 180, 59–62. 10.1007/s11845-010-0629-5 21052862

[B15] Callaerts-Vegh Z. Ahmed T. Vermaercke B. Marynen P. Balschun D. Froyen G.. (2015). Nxf7 deficiency impairs social exploration and spatio-cognitive abilities as well as hippocampal synaptic plasticity in mice. Front. Behav. Neurosci. 9:179. 10.3389/fnbeh.2015.00179 26217206 PMC4498129

[B16] Chatterson L. C. Leswick D. A. Fladeland D. A. Hunt M. M. Webster S. Lim H. (2014). Fetal shielding combined with state of the art CT dose reduction strategies during maternal chest CT. Eur. J. Radiol. 83, 1199–1204. 10.1016/j.ejrad.2014.04.020 24838282

[B17] Cipollone D. Carsetti R. Tagliani A. Rosado M. M. Borgiani P. Novelli G.. (2009). Folic acid and methionine in the prevention of teratogen-induced congenital defects in mice. Cardiovasc. Pathol. 18, 100–109. 10.1016/j.carpath.2008.02.007 18417366

[B18] Craenen K. Verslegers M. Baatout S. Abderrafi Benotmane M. (2020a). An appraisal of folates as key factors in cognition and ageing-related diseases. Crit. Rev. Food Sci. Nutr. 60, 722–739. 10.1080/10408398.2018.1549017 30729795

[B19] Craenen K. Verslegers M. Buset J. Baatout S. Moons L. Benotmane M. A. (2017). A detailed characterization of congenital defects and mortality following moderate X-ray doses during neurulation. Birth Defects Res. 110, 467–482. 10.1002/bdr2.1161 29193908

[B20] Craenen K. Verslegers M. Craeghs L. Quintens R. Janssen A. Coolkens A.. (2020b). Abnormal retinal pigment epithelium melanogenesis as a major determinant for radiation-induced congenital eye defects. Reprod. Toxicol. 91, 59–73. 10.1016/j.reprotox.2019.10.002 31705956

[B21] Crider K. S. Yang T. P. Berry R. J. Bailey L. B. (2012). Folate and DNA methylation: a review of molecular mechanisms and the evidence for folate's role. Adv. Nutr. 3, 21–38. 10.3945/an.111.000992 22332098 PMC3262611

[B22] Curhan G. C. Willett W. C. Rimm E. B. Spiegelman D. Ascherio A. L. Stampfer M. J. (1996). Birth weight and adult hypertension, diabetes mellitus, and obesity in US men. Circulation 94, 3246–50. 10.1161/01.CIR.94.12.3246 8989136

[B23] Czeizel A. E. Bártfai Z. Bánhidy F. (2011). Primary prevention of neural-tube defects and some other congenital abnormalities by folic acid and multivitamins: history, missed opportunity and tasks. Ther. Adv. drug Saf. 2, 173–188. 10.1177/2042098611411358 25083211 PMC4110861

[B24] De Groef L. Andries L. Siwakoti A. Geeraerts E. Bollaerts I. Noterdaeme L.. (2016). Aberrant collagen composition of the trabecular meshwork results in reduced aqueous humor drainage and elevated IOP in MMP-9 null mice. Investig. Opthalmol. Vis. Sci. 57:5984. 10.1167/iovs.16-19734 27820954 PMC5102567

[B25] Dennery P. A. (2007). Effects of oxidative stress on embryonic development. Birth Defects Res. C Embryo Today Rev. 81, 155–162. 10.1002/bdrc.20098 17963268

[B26] Di Majo V. Ballardin E. Metalli P. (1981). Comparative effects of fission neutron and X irradiation on 7.5-day mouse embryos. Radiat. Res. 87, 145–58. 10.2307/3575548 7255668

[B27] Ferm V. H. Hanlon D. P. (1986). Arsenate-induced neural tube defects not influenced by constant rate administration of folic acid. Pediatr. Res. 20, 761–762. 10.1203/00006450-198608000-00012 3737289

[B28] Fujimori K. Kyozuka H. Yasuda S. Goto A. Yasumura S. Ota M. (2014). Pregnancy and birth survey after the Great East Japan earthquake and Fukushima Daiichi nuclear power plant accident in Fukushima prefecture. Fukushima J. Med. Sci. 60, 75–81. 10.5387/fms.2014-9 25030719

[B29] Gekas J. Li B. Kamnasaran D. (2010). Current perspectives on the etiology of agnathia-otocephaly. Eur. J. Med. Genet. 53, 358–366. 10.1016/j.ejmg.2010.09.002 20849990

[B30] Godwin K. A. Sibbald B. Bedard T. Kuzeljevic B. Lowry R. B. Arbour L. (2008). Changes in frequencies of select congenital anomalies since the onset of folic acid fortification in a Canadian birth defect registry. Can. J. Public Health 99, 271–5. 10.1007/BF03403753 18767269 PMC6975565

[B31] Gray J. D. Ross M. E. (2009). Mechanistic insights into folate supplementation from Crooked tail and other NTD-prone mutant mice. Birth Defects Res. A Clin. Mol. Teratol. 85, 314–321. 10.1002/bdra.20542 19067399 PMC2811164

[B32] Harris M. J. (2009). Insights into prevention of human neural tube defects by folic acid arising from consideration of mouse mutants. Birth Defects Res. A Clin. Mol. Teratol. 85, 331–339. 10.1002/bdra.20552 19117321

[B33] Herman S. Delio M. Morrow B. Samanich J. (2012). Agnathia–otocephaly complex: a case report and examination of the OTX2 and PRRX1 genes. Gene 494, 124–129. 10.1016/j.gene.2011.11.033 22198066

[B34] Heyer B. S. MacAuley A. Behrendtsen O. Werb Z. (2000). Hypersensitivity to DNA damage leads to increased apoptosis during early mouse development. Genes Dev. 14, 2072–2084. 10.1101/gad.14.16.2072 10950870 PMC316856

[B35] Hindin R. Brugge D. Panikkar B. (2005). Teratogenicity of depleted uranium aerosols: a review from an epidemiological perspective. Environ. Heal. 4:17. 10.1186/1476-069X-4-17 16124873 PMC1242351

[B36] Imbard A. Benoist J.-F. Blom H. J. (2013). Neural tube defects, folic acid and methylation. Int. J. Environ. Res. Public Health 10, 4352–4389. 10.3390/ijerph10094352 24048206 PMC3799525

[B37] Jagtap S. V. Saini N. Jagtap S. Saini S. (2015). Otocephaly: Agnathia- Microstomia-Synotia Syndrome- A Rare Congenital Anomaly. J. Clin. Diagn. Res. 9, ED03-4. 10.7860/JCDR/2015/13636.6444 26500912 PMC4606241

[B38] Jarmonenko S. P. (1988). Radiobiology of Humans and Animals. Mir Publishers. Available online at: https://books.google.be/books/about/Radiobiology_of_Humans_and_Animals.html?id=qo8tAAAACAAJandredir_esc=y (accessed October 10, 2018).

[B39] Johnsson I. W. Haglund B. Ahlsson F. Gustafsson J. (2015). A high birth weight is associated with increased risk of type 2 diabetes and obesity. Pediatr. Obes. 10, 77–83. 10.1111/ijpo.230 24916852

[B40] Juriloff D. M. (1985). Prevention of the eye closure defect inlgMl/lgMl fetal mice by thyroxine. Teratology 32, 73–86. 10.1002/tera.1420320111 4035594

[B41] Kappen C. (2013). Modeling anterior development in mice: diet as modulator of risk for neural tube defects. Am. J. Med. Genet. C Semin. Med. Genet. 163, 333–356. 10.1002/ajmg.c.31380 PMC4149464 24124024

[B42] Kim S. H. R. Lee J. H. Oh H. Kim S. H. R. Lee C. S. Jo S. K.. (2001). Dependence of malformation upon gestational age and exposed dose of gamma radiation. J. Radiat. Res. 42, 255–64. 10.1269/jrr.42.255 11840642

[B43] Latif-Hernandez A. Shah D. Ahmed T. Lo A. C. Callaerts-Vegh Z. Van der Linden A.. (2016). Quinolinic acid injection in mouse medial prefrontal cortex affects reversal learning abilities, cortical connectivity and hippocampal synaptic plasticity. Sci. Rep. 6:36489. 10.1038/srep36489 27819338 PMC5098239

[B44] Lazarus E. Debenedectis C. North D. Spencer P. K. Mayo-Smith W. W. (2009). Utilization of imaging in pregnant patients: 10-year review of 5270 examinations in 3285 patients−1997-2006. Radiology 251, 517–524. 10.1148/radiol.2512080736 19293204

[B45] Lehmkuhl A. M. Dirr E. R. Fleming S. M. (2014). Olfactory assays for mouse models of neurodegenerative disease. J. Vis. Exp. 90:e51804. 10.3791/51804 25177842 PMC4827975

[B46] Levin M. (2005). Left-right asymmetry in embryonic development: a comprehensive review. Mech. Dev. 122, 3–25. 10.1016/j.mod.2004.08.006 15582774

[B47] Li J. M. Qu P. F. Dang S. N. Li S. S. Bai R. H. Qin B. W.. (2016). Effect of folic acid supplementation in childbearing aged women during pregnancy on neonate birth weight in Shaanxi province. Zhonghua Liu Xing Bing Xue Za Zhi 37, 1017–20. 10.3760/cma.j.issn.0254-6450.2016.07.022 27453116

[B48] Lin C.-W. Lo C.-P. Tu M.-C. (2018). Horizontal gaze palsy with progressive scoliosis: a case report with magnetic resonance tractography and electrophysiological study. BMC Neurol. 18:75. 10.1186/s12883-018-1081-9 29843650 PMC5972445

[B49] Lindner M. D. Plone M. A. Schallert T. Emerich D. F. (1997). Blind rats are not profoundly impaired in the reference memory Morris water maze and cannot be clearly discriminated from rats with cognitive deficits in the cued platform task. Brain Res. Cogn. Brain Res. 5, 329–33. 10.1016/S0926-6410(97)00006-2 9197520

[B50] Lo A. C. Callaerts-Vegh Z. Nunes A. F. Rodrigues C. M. P. D'Hooge R. (2013). Tauroursodeoxycholic acid (TUDCA) supplementation prevents cognitive impairment and amyloid deposition in APP/PS1 mice. Neurobiol. Dis. 50, 21–29. 10.1016/j.nbd.2012.09.003 22974733

[B51] Maestro-de-las-Casas C. Pérez-Miguelsanz J. López-Gordillo Y. Maldonado E. Partearroyo T. Varela-Moreiras G.. (2013). Maternal folic acid-deficient diet causes congenital malformations in the mouse eye. Birth Defects Res. A Clin. Mol. Teratol. 97, 587–596. 10.1002/bdra.23176 24078476

[B52] Mangano J. Sherman J. D. (2015). Changes in congenital anomaly incidence in West Coast and Pacific States (USA) after arrival of Fukushima fallout. Open J. Pediatr. 05, 76–89. 10.4236/ojped.2015.51013

[B53] Martin L. M. Marples B. Lynch T. H. Hollywood D. Marignol L. (2014). Exposure to low dose ionising radiation: Molecular and clinical consequences. Cancer Lett. 349, 98–106. 10.1016/j.canlet.2013.12.015 24983100

[B54] Mettler F. A. Bhargavan M. Faulkner K. Gilley D. B. Gray J. E. Ibbott G. S.. (2009). Radiologic and nuclear medicine studies in the United States and worldwide: frequency, radiation dose, and comparison with other radiation sources−1950-2007. Radiology 253, 520–531. 10.1148/radiol.2532082010 19789227

[B55] Modat M. Ridgway G. R. Taylor Z. A. Lehmann M. Barnes J. Hawkes D. J.. (2010). Fast free-form deformation using graphics processing units. Comput. Methods Programs Biomed. 98, 278–284. 10.1016/j.cmpb.2009.09.002 19818524

[B56] Moore W. Bonvento M. J. Lee D. Dunkin J. Bhattacharji P. (2015). Reduction of fetal dose in computed tomography using anterior shields. J. Comput. Assist. Tomogr. 39, 298–300. 10.1097/RCT.0000000000000190 25786095

[B57] Moster S. J. Moster M. L. Scannell Bryan M. Sergott R. C. (2016). Retinal Ganglion Cell and inner plexiform layer loss correlate with visual acuity loss in LHON: a longitudinal, segmentation OCT analysis. Investig. Opthalmol. Vis. Sci. 57:3872. 10.1167/iovs.15-17328 27459664

[B58] Muralidharan P. Sarmah S. Marrs J. A. (2015). Zebrafish retinal defects induced by ethanol exposure are rescued by retinoic acid and folic acid supplement. Alcohol 49, 149–163. 10.1016/j.alcohol.2014.11.001 25541501 PMC4339401

[B59] Neel J. Schull W. J. (1956). Effect of Exposure to the Atomic Bombs on Pregnancy Termination in Hiroshima and Nagasaki. Washington, DC: National Academies Press. 25077208

[B60] Owrangi A. M. Roberts D. A. Covington E. L. Hayman J. A. Masi K. M. Lee C. (2016). Revisiting fetal dose during radiation therapy: evaluating treatment techniques and a custom shield. J. Appl. Clin. Med. Phys. 17, 34–46. 10.1120/jacmp.v17i5.6135 PMC5874082 27685109

[B61] Oyama K. Sugimura Y. Murase T. Uchida A. Hayasaka S. Oiso Y. (2009). Folic acid prevents congenital malformations in the offspring of diabetic mice. Endocr. J. 56, 29–37. 10.1507/endocrj.K08E-180 18781038

[B62] Pampfer S. Streffer C. (1988). Prenatal death and malformations after irradiation of mouse zygotes with neutrons or X-rays. Teratology 37, 599–607. 10.1002/tera.1420370609 3400074

[B63] Paranjothy S. Broughton H. Evans A. Huddart S. Drayton M. Jefferson R.. (2012). The role of maternal nutrition in the aetiology of gastroschisis: an incident case-control study. Int. J. Epidemiol. 41, 1141–1152. 10.1093/ije/dys092 22798661

[B64] Phillips J. B. Youmans P. W. Muheim R. Sloan K. A. Landler L. Painter M. S.. (2013). Rapid learning of magnetic compass direction by C57BL/6 mice in a 4-armed “plus” water maze. PLoS ONE 8:e73112. 10.1371/journal.pone.0073112 24023673 PMC3758273

[B65] Plummer G. (1952). Anomalies occurring in children exposed in utero to the atomic bomb in Hiroshima. Pediatrics 10, 687–693. Available online at: http://pediatrics.aappublications.org/content/10/6/687 (accessed August 27, 2017). 13003418

[B66] Portmann T. Yang M. Mao R. Panagiotakos G. Ellegood J. Dolen G.. (2014). Behavioral abnormalities and circuit defects in the basal ganglia of a mouse model of 16p11.2 deletion syndrome. Cell Rep. 7, 1077–1092. 10.1016/j.celrep.2014.03.036 24794428 PMC4251471

[B67] Qiu M.-H. Vetrivelan R. Fuller P. M. Lu J. (2010). Basal ganglia control of sleep-wake behavior and cortical activation. Eur. J. Neurosci. 31, 499–507. 10.1111/j.1460-9568.2009.07062.x 20105243 PMC3928571

[B68] Ramakrishnan U. Nguyen P. H. Gonzalez-Casanova I. Pham H. Hao W. Nguyen H.. (2016). Neither preconceptional weekly multiple micronutrient nor iron-folic acid supplements affect birth size and gestational age compared with a folic acid supplement alone in rural vietnamese women: a randomized controlled trial. J. Nutr. 146, 1445S−1452S. 10.3945/jn.115.223420 27281806

[B69] Regnier C. H. Masson R. Kedinger V. Textoris J. Stoll I. Chenard M.-P.. (2002). Impaired neural tube closure, axial skeleton malformations, and tracheal ring disruption in TRAF4-deficient mice. Proc. Natl. Acad. Sci. U.S.A. 99, 5585–5590. 10.1073/pnas.052124799 11943846 PMC122813

[B70] Reisz J. A. Bansal N. Qian J. Zhao W. Furdui C. M. (2014). Effects of ionizing radiation on biological molecules–mechanisms of damage and emerging methods of detection. Antioxid. Redox Signal. 21, 260–292. 10.1089/ars.2013.5489 24382094 PMC4060780

[B71] Russell L. B. (1950). X-ray induced developmental abnormalities in the mouse and their use in the analysis of embryological patterns. I. External and gross visceral changes. J. Exp. Zool. 114, 545–601. 10.1002/jez.1401140307

[B72] Russell L. B. (1956). X-ray-induced developmental abnormalities in the mouse and their use in the analysis of embryological patterns. II. Abnormalities of the vertebral column and thorax. J. Exp. Zool. 131, 329–395. 10.1002/jez.1401310308

[B73] Scherb H. H. Mori K. Hayashi K. (2016). Increases in perinatal mortality in prefectures contaminated by the Fukushima nuclear power plant accident in Japan. Medicine 95:e4958. 10.1097/MD.0000000000004958 27661055 PMC5044925

[B74] Sergouniotis P. I. Urquhart J. E. Williams S. G. Bhaskar S. S. Black G. C. Lovell S. C.. (2015). Agnathia-otocephaly complex and asymmetric velopharyngeal insufficiency due to an in-frame duplication in OTX2. J. Hum. Genet. 60, 199–202. 10.1038/jhg.2014.122 25589041

[B75] Shaw G. M. Carmichael S. L. Laurent C. Louik C. Finnell R. H. Lammer E. J.. (2007). Nutrient intakes in women and risks of anophthalmia and microphthalmia in their offspring. Birth Defects Res. A Clin. Mol. Teratol. 79, 708–713. 10.1002/bdra.20398 17847120

[B76] Sinding C. Valadier F. Al-Hassani V. Feron G. Tromelin A. Kontaris I. (2017). New determinants of olfactory habituation. Sci. Rep. 7:41047 10.1038/srep41047 28120877 PMC5264389

[B77] Smith R. S. Roderick T. H. Sundberg J. P. (1994). Microphthalmia and associated abnormalities in inbred black mice. Lab. Anim. Sci. 44, 551–60. Available online at: http://www.ncbi.nlm.nih.gov/pubmed/7898027 (accessed August 25, 2016). 7898027

[B78] Stroobants S. Leroy T. Eckhardt M. Aerts J.-M. Berckmans D. D'Hooge R. (2008). Early signs of neurolipidosis-related behavioural alterations in a murine model of metachromatic leukodystrophy. Behav. Brain Res. 189, 306–316. 10.1016/j.bbr.2008.01.008 18336930

[B79] Strupp M. Kremmyda O. Adamczyk C. Böttcher N. Muth C. Yip C. W.. (2014). Central ocular motor disorders, including gaze palsy and nystagmus. J. Neurol. 261(Suppl. 2), S542–S558. 10.1007/s00415-014-7385-9 25145891 PMC4141156

[B80] Tamura T. Picciano M. F. (2006). Folate and human reproduction. Am. J. Clin. Nutr. 83, 993–1016. 10.1093/ajcn/83.5.993 16685040

[B81] Treloar H. B. Miller A. M. Ray A. Greer C. A. (2010). Development of the Olfactory System. CRC Press/Taylor and Francis Available online at: http://www.ncbi.nlm.nih.gov/pubmed/21882426 (accessed September 16, 2018). 21882426

[B82] Tustison N. J. Avants B. B. Cook P. A. Yuanjie Z.heng Egan A. Yushkevich P. A.. (2010). N4ITK: improved N3 bias correction. IEEE Trans. Med. Imaging 29, 1310–1320. 10.1109/TMI.2010.2046908 20378467 PMC3071855

[B83] Tyll S. Budinger E. Noesselt T. (2011). Thalamic influences on multisensory integration. Commun. Integr. Biol. 4, 378–381. 10.4161/cib.15222 21966551 PMC3181501

[B84] Van Hove I. Lefevere E. De Groef L. Sergeys J. Salinas-Navarro M. Libert C.. (2016). MMP-3 deficiency alleviates endotoxin-induced acute inflammation in the posterior eye segment. Int. J. Mol. Sci. 17:1825. 10.3390/ijms17111825 27809288 PMC5133826

[B85] Verma A. S. Fitzpatrick D. R. (2007). Anophthalmia and microphthalmia. Orphanet J. Rare Dis. 2:47. 10.1186/1750-1172-2-47 18039390 PMC2246098

[B86] Verreet T. Quintens R. Van Dam D. Verslegers M. Tanori M. Casciati A.. (2015). A multidisciplinary approach unravels early and persistent effects of X-ray exposure at the onset of prenatal neurogenesis. J. Neurodev. Disord. 7:3. 10.1186/1866-1955-7-3 26029273 PMC4448911

[B87] Verreet T. Rangarajan J. R. Quintens R. Verslegers M. Lo A. C. Govaerts K.. (2016a). persistent impact of *in utero* irradiation on mouse brain structure and function characterized by MR imaging and behavioral analysis. Front. Behav. Neurosci. 10:83. 10.3389/fnbeh.2016.00083 27199692 PMC4854899

[B88] Verreet T. Verslegers M. Quintens R. Baatout S. Benotmane M. A. (2016b). Current evidence for developmental, structural, and functional brain defects following prenatal radiation exposure. Neural Plast. 2016:1243527. 10.1155/2016/1243527 27382490 PMC4921147

[B89] Vorhees C. V. Williams M. T. (2014). Assessing spatial learning and memory in rodents. ILAR J. 55, 310–332. 10.1093/ilar/ilu013 25225309 PMC4240437

[B90] Wentzel P. Eriksson U. J. (2005). A diabetes-like environment increases malformation rate and diminishes prostaglandin E2 in rat embryos: reversal by administration of vitamin E and folic acid. Birth Defects Res. A Clin. Mol. Teratol. 73, 506–511. 10.1002/bdra.20145 15959876

[B91] Wertelecki W. Koerblein A. Ievtushok B. Zymak-Zakutnia N. Komov O. Kuznietsov I.. (2016). Elevated congenital anomaly rates and incorporated cesium-137 in the Polissia region of Ukraine. Birth Defects Res. A. Clin. Mol. Teratol. 106, 194–200. 10.1002/bdra.23476 26871487

[B92] Wiens D. DeSoto M. (2017). Is high folic acid intake a risk factor for autism?—a review. Brain Sci. 7:149. 10.3390/brainsci7110149 29125540 PMC5704156

[B93] Xu Y. Li Y. Tang Y. Wang J. Shen X. Long Z.. (2006). The maternal combined supplementation of folic acid and Vitamin B12 suppresses ethanol-induced developmental toxicity in mouse fetuses. Reprod. Toxicol. 22, 56–61. 10.1016/j.reprotox.2005.12.004 16439097

[B94] Yanaguita M. Y. Gutierrez C. M. Ribeiro C. N. M. Lima G. A. Machado H. R. Peres L. C. (2007). Pregnancy outcome in ethanol-treated mice with folic acid supplementation in saccharose. Childs Nerv. Syst. 24, 99–104. 10.1007/s00381-007-0427-1 17619885

[B95] Yang M. Abrams D. N. Zhang J. Y. Weber M. D. Katz A. M. Clarke A. M.. (2012). Low sociability in BTBR T+tf/J mice is independent of partner strain. Physiol. Behav. 107, 649–662. 10.1016/j.physbeh.2011.12.025 22245067 PMC3330157

[B96] Yang M. Crawley J. N. (2009). Simple behavioral assessment of mouse olfaction, in Current Protocols in Neuroscience (Hoboken, NJ: John Wiley and Sons, Inc.) 8.24.1–8.24.12. 10.1002/0471142301.ns0824s48 PMC2753229 19575474

[B97] Yang W. Carmichael S. L. Shaw G. M. (2016). Folic acid fortification and prevalences of neural tube defects, orofacial clefts, and gastroschisis in California, 1989 to 2010. Birth Defects Res. A Clin. Mol. Teratol. 106, 1032–1041. 10.1002/bdra.23514 27191125

[B98] Young A. D. Phipps D. E. Astroff A. B. (2000). Large-scale double-staining of rat fetal skeletons using Alizarin Red S and Alcian Blue. Teratology 61, 273–276. 10.1002/(SICI)1096-9926(200004)61:4<273::AID-TERA5<3.0.CO;2-2 10716745

