# Supplementary figures and images for: Folic Acid Fortification Prevents Morphological and Behavioral Consequences of X-Ray Exposure During Neurulation
Source: Front Behav Neurosci. 2021 Jan 8;14:609660. doi: 10.3389/fnbeh.2020.609660 (PMC7820780; doi:10.3389/fnbeh.2020.609660)

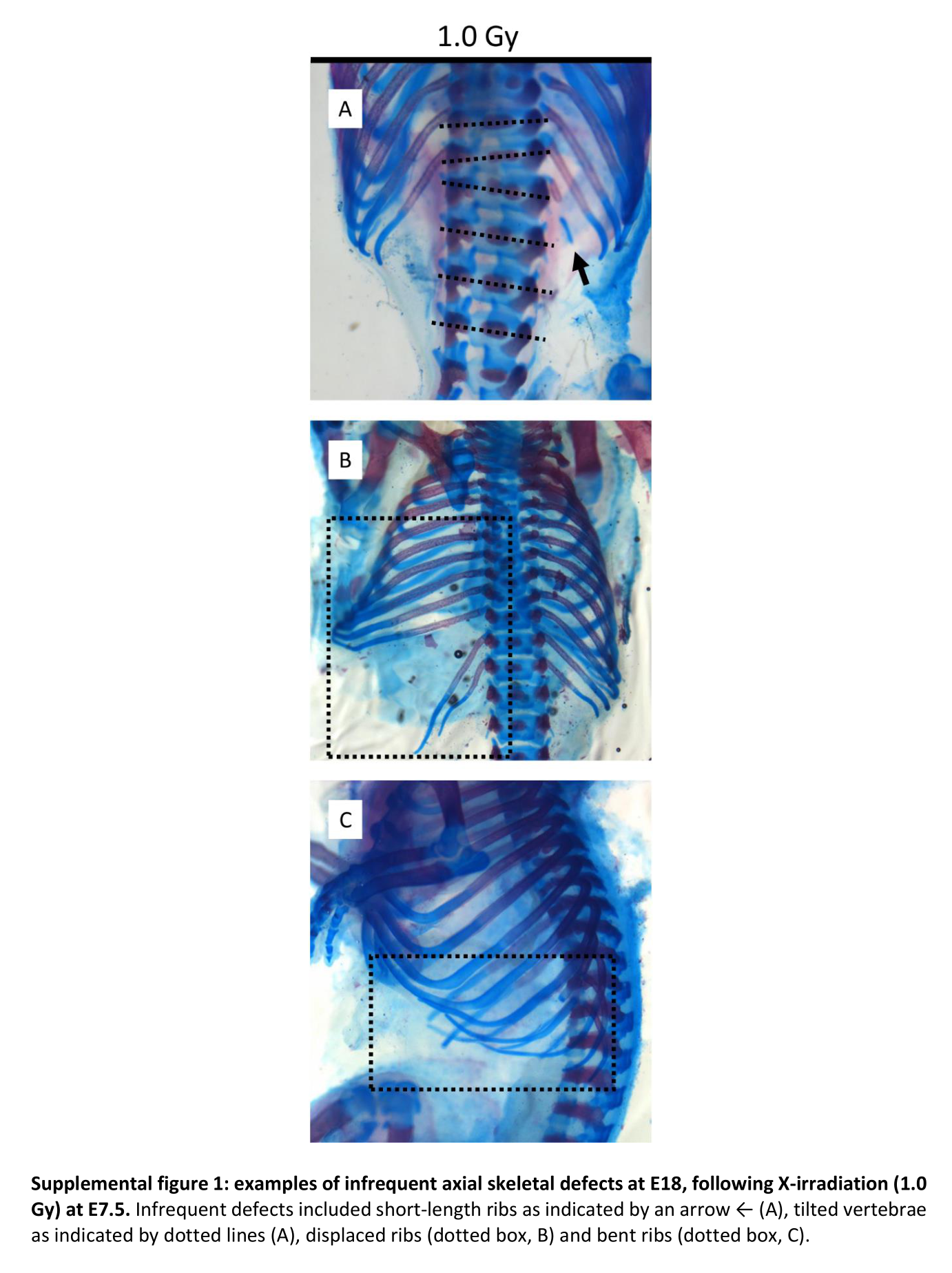

Supplement: Supplementary file 1 [file Image_1.TIFF]

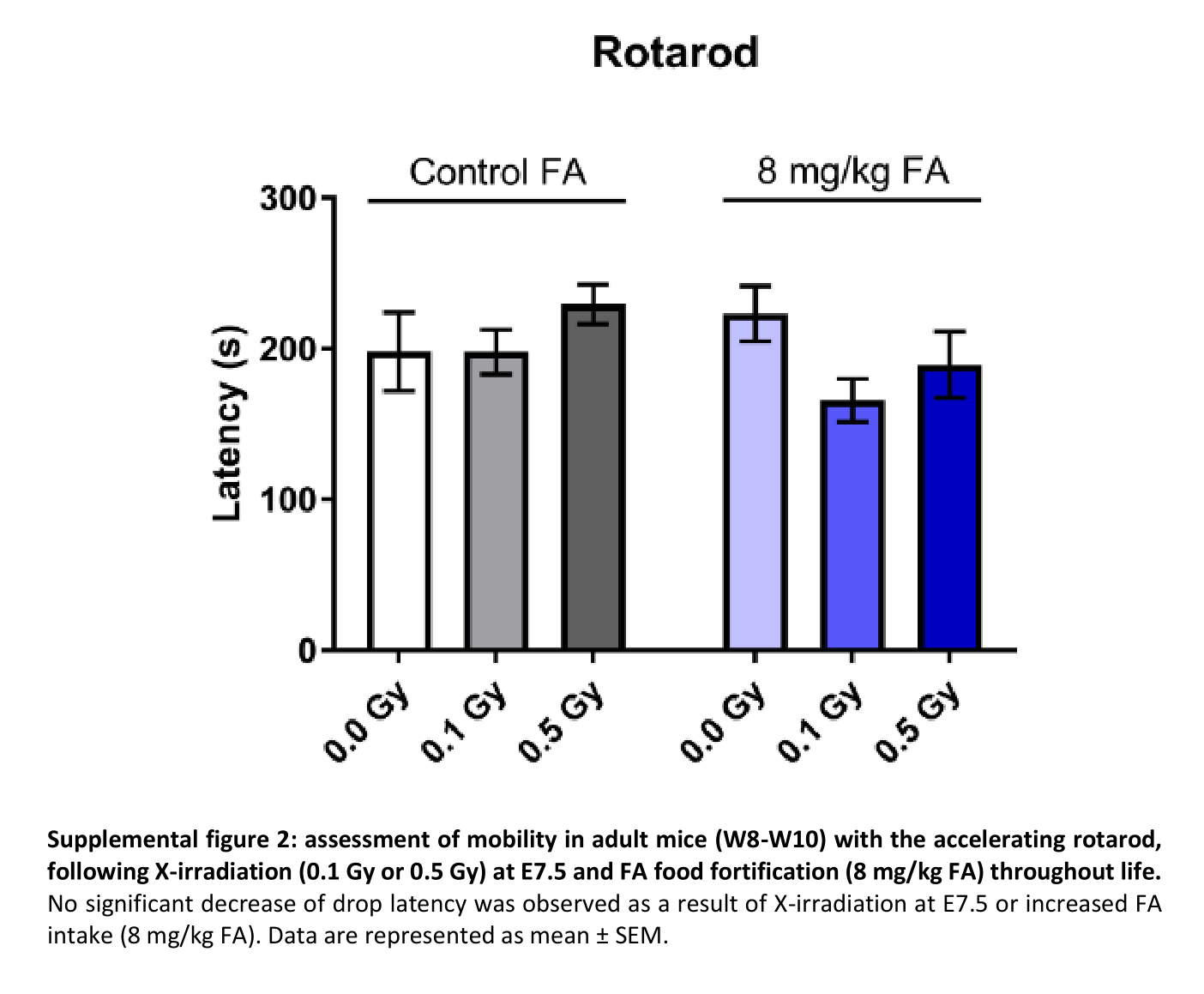

Supplement: Supplementary file 2 [file Image_2.TIFF]

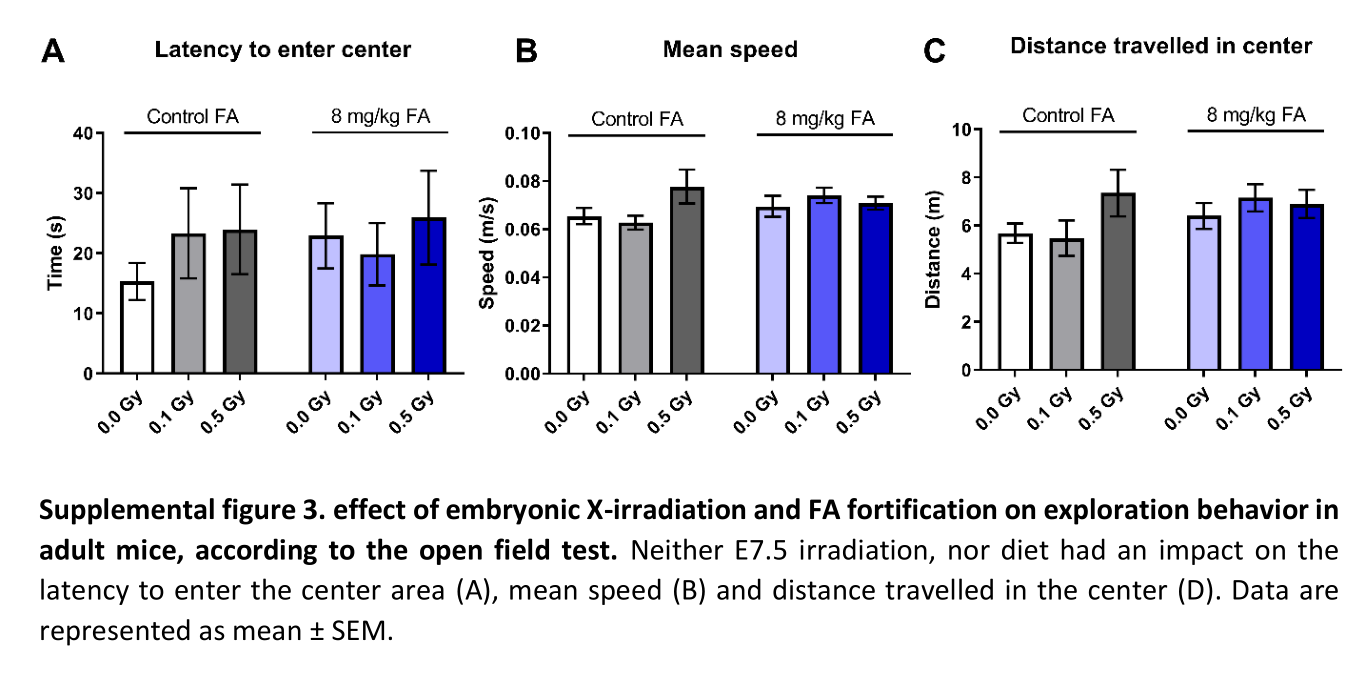

Supplement: Supplementary file 3 [file Image_3.TIFF]

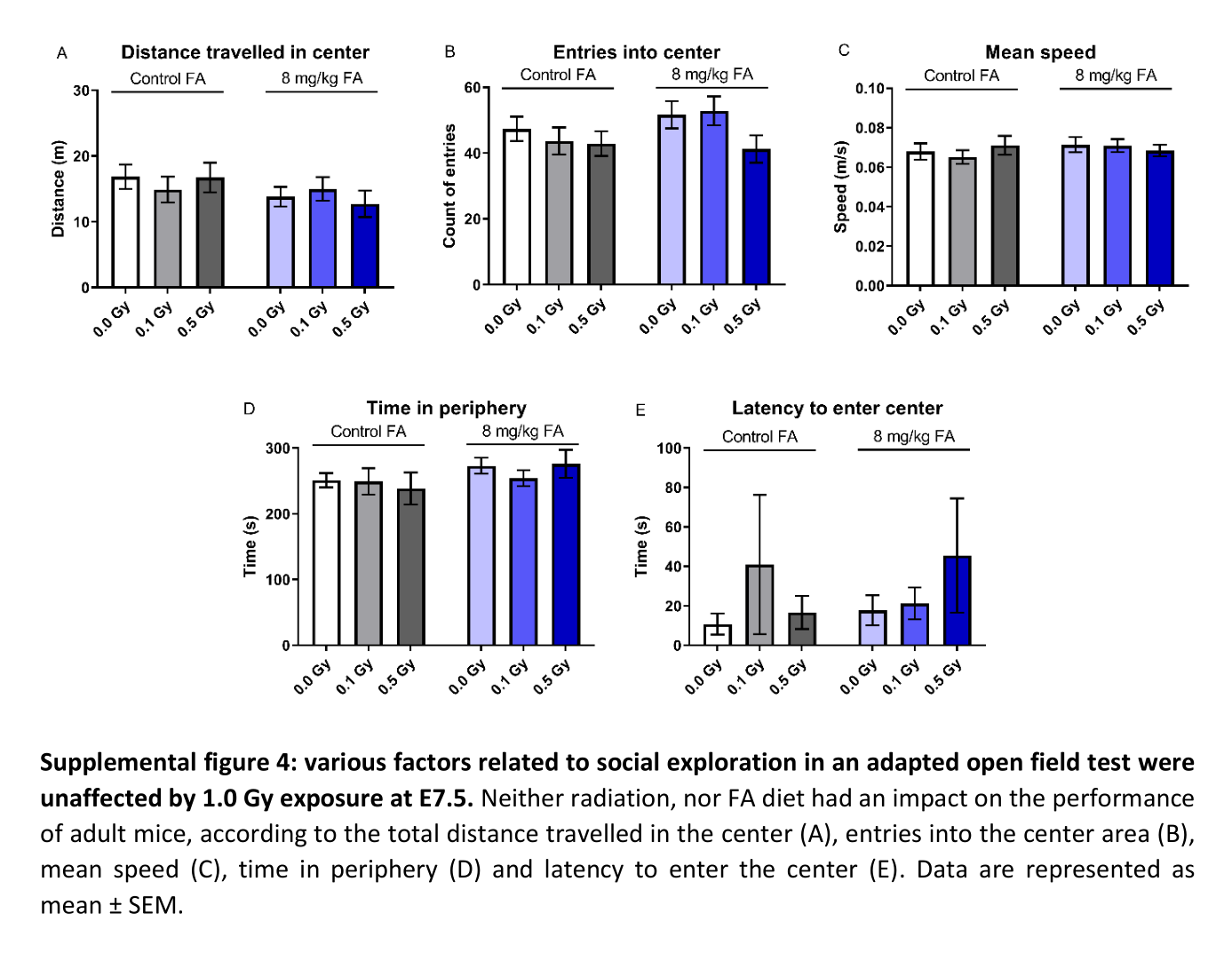

Supplement: Supplementary file 4 [file Image_4.TIFF]

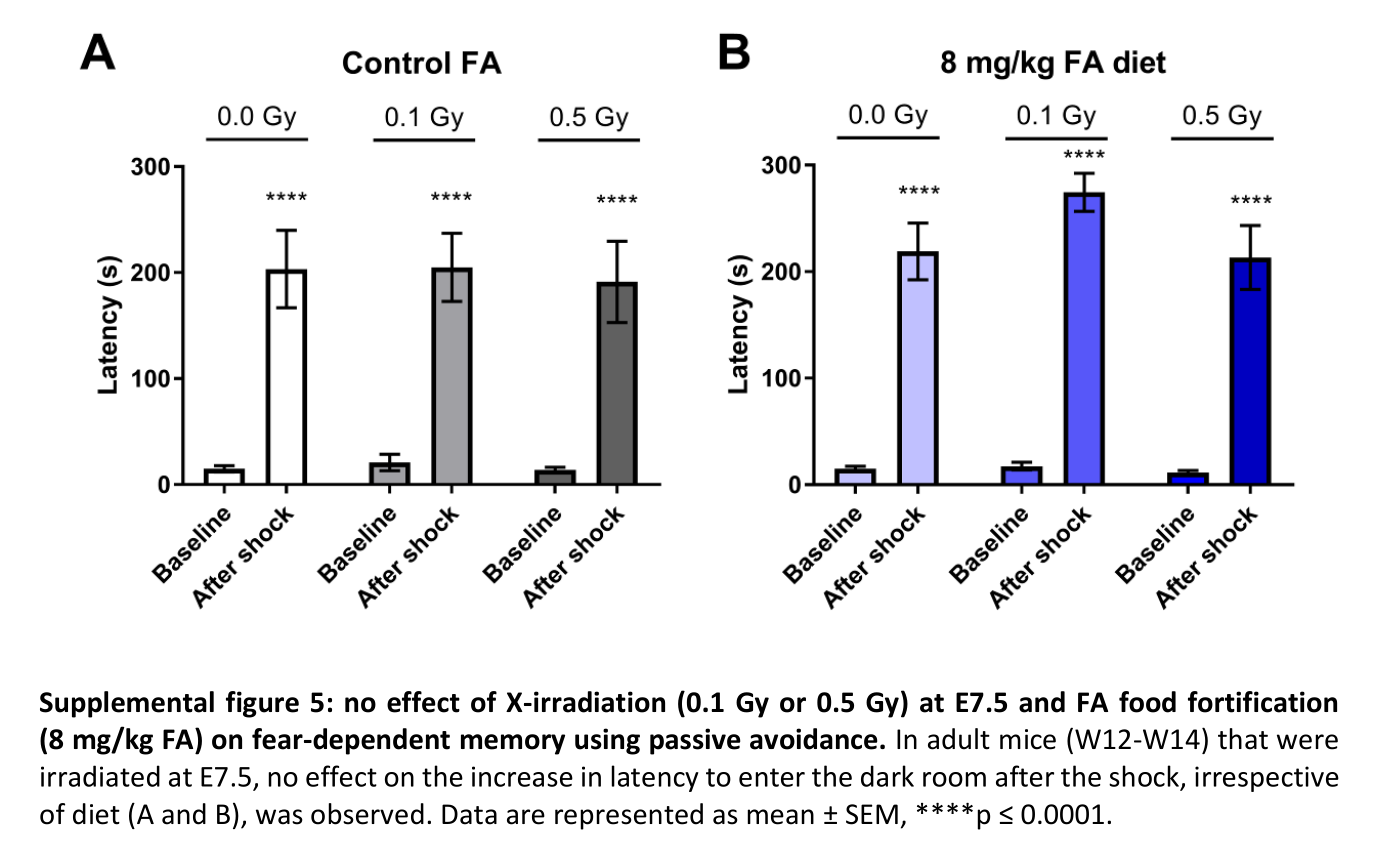

Supplement: Supplementary file 5 [file Image_5.TIFF]
