# Supplementary material for: Folic Acid Fortification Prevents Morphological and Behavioral Consequences of X-Ray Exposure During Neurulation
Source: Front Behav Neurosci. 2021 Jan 8;14:609660. doi: 10.3389/fnbeh.2020.609660 (PMC7820780; doi:10.3389/fnbeh.2020.609660)
Supplement: Supplementary file 6 [file Table_1.DOCX]

| Supplemental table 1. Absolute volumes (mm^3^) of brain regions in adult mice, according to MRI measurements and atlas-based segmentation, following irradiation at E7.5. | | | | | | | | | |
| --- | --- | --- | --- | --- | --- | --- | --- | --- | --- |
|  | Control diet | | | High FA diet | | |  | | |
|  | 0.0 Gy (N = 9) | 0.1 Gy (N=12) | 0.5 Gy (N = 5) | 0.0 Gy (N = 5) | 0.1 Gy (N = 7) | 0.5 Gy (N = 5) | Interaction F&P | Radiation F&P | Diet F&P |
| Total brain | 426.69 ± 8.70 | 420.06 ± 7.86 | 408.19 ± 13.97 | 411.42 ± 8.46 | 412.75 ± 8.54 | 413.89 ± 7.64 | F (2, 37) = 1,147  P=0,3286 | F (2, 37) = 0,6797  P=0,5130 | F (1, 37) = 1,12  P=0,2968 |
| Olfactory system | 36.32 ± 0.53 | 36.24 ± 0.49 | 36.23 ± 1.43 | 37.22 ± 1.09 | 35.55 ± 0.71 | 34.73 ± 0.78 | F (2, 37) = 1,093  P=0,3458 | F (2, 37) = 1,275  P=0,2915 | F (1, 37) = 0,4443  P=0,5092 |
| Frontal cortex | 15.75 ± 0.31 | 15.54 ± 0.29 | 15.05 ± 0.66 | 16.01 ± 0.29 | 15.39 ± 0.25 | 14.97 ± 0.29 | F (2, 37) = 0,1997  P=0,8199 | F (2, 37) = 2,445  P=0,1006 | F (1, 37) = 0,0007355  P=0,9785 |
| Corpus callosum | 10.58 ± 0.30 | 10.11 ± 0.22 | 9.96 ± 0.32 | 9.81 ± 0.42 | 9.96 ± 0.24 | 10.20 ± 0.25 | F (2, 37) = 1,292  P=0,2869 | F (2, 37) = 0,1521  P=0,8594 | F (1, 37) = 0,8236  P=0,3700 |
| Amygdala | 12.15 ± 0.29 | 12.09 ± 0.24 | 11.62 ± 0.23 | 11.25 ± 0.23 | 12.00 ± 0.18 | 11.93 ± 0.27 | F (2, 37) = 2,248  P=0,1199 | F (2, 37) = 1,002  P=0,3767 | F (1, 37) = 0,9626  P=0,3329 |
| Cerebellum | 45.78 ± 0.82 | 46.03 ± 0.83 | 45.73 ± 1.21 | 45.47 ± 0.60 | 44.90 ± 0.82 | 45.06 ± 0.56 | F (2, 37) = 0,1135  P=0,8930 | F (2, 37) = 0,03043  P=0,9701 | F (1, 37) = 0,8335  P=0,3672 |
| Corpora quadrigemina | 15.68 ± 0.46 | 14.99 ± 0.25 | 15.12 ± 0.27 | 14.85 ± 0.54 | 14.66 ± 0.17 | 14.87 ± 0.43 | F (2, 37) = 0,3271  P=0,7231 | F (2, 37) = 0,7512  P=0,4789 | F (1, 37) = 2,253  P=0,1419 |
| According to MRI scan at adult age, neither irradiation at E7.5 with 0.1 Gy or 0.5 Gy, nor diet had any effect on the volume of the total brain, olfactory system, frontal cortex, corpus callosum, amygdala, cerebellum and corpora quadrigemina. | | | | | | | | | |
